# The bright future of nanotechnology in lymphatic system imaging and imaging-guided surgery

**DOI:** 10.1186/s12951-021-01232-5

**Published:** 2022-01-06

**Authors:** Shaolong Qi, Xinyu Wang, Kun Chang, Wenbin Shen, Guocan Yu, Jianshi Du

**Affiliations:** 1 Key Laboratory & Engineering Laboratory of Lymphatic Surgery Jilin Province, China-Japan Union Hospital of Jilin University, Changchun, 130031 People’s Republic of China; 2 Key Laboratory of Bioorganic Phosphorus Chemistry & Chemical Biology, Department of Chemistry, Tsinghua University, Beijing, 100084 People’s Republic of China; 3 Department of Lymphology, Beijing Shijitan Hospital, Capital Medical University, Beijing, 100038 People’s Republic of China

**Keywords:** Lymphatic system imaging, Nanotechnology, Lymphoscintigraphy, Multimodal imaging, Imaging-guided surgery

## Abstract

Lymphatic system is identified the second vascular system after the blood circulation in mammalian species, however the research on lymphatic system has long been hampered by the lack of comprehensive imaging modality. Nanomaterials have shown the potential to enhance the quality of lymphatic imaging due to the unparalleled advantages such as the specific passive targeting and efficient co-delivery of cocktail to peripheral lymphatic system, ease molecular engineering for precise active targeting and prolonged retention in the lymphatic system of interest. Multimodal lymphatic imaging based on nanotechnology provides a complementary means to understand the kinetics of lymphoid tissues and quantify its function. In this review, we introduce the established approaches of lymphatic imaging used in clinic and summarize their strengths and weaknesses, and list the critical influence factors on lymphatic imaging. Meanwhile, the recent developments in the field of pre-clinical lymphatic imaging are discussed to shed new lights on the design of new imaging agents, the improvement of delivery methods and imaging-guided surgery strategies.

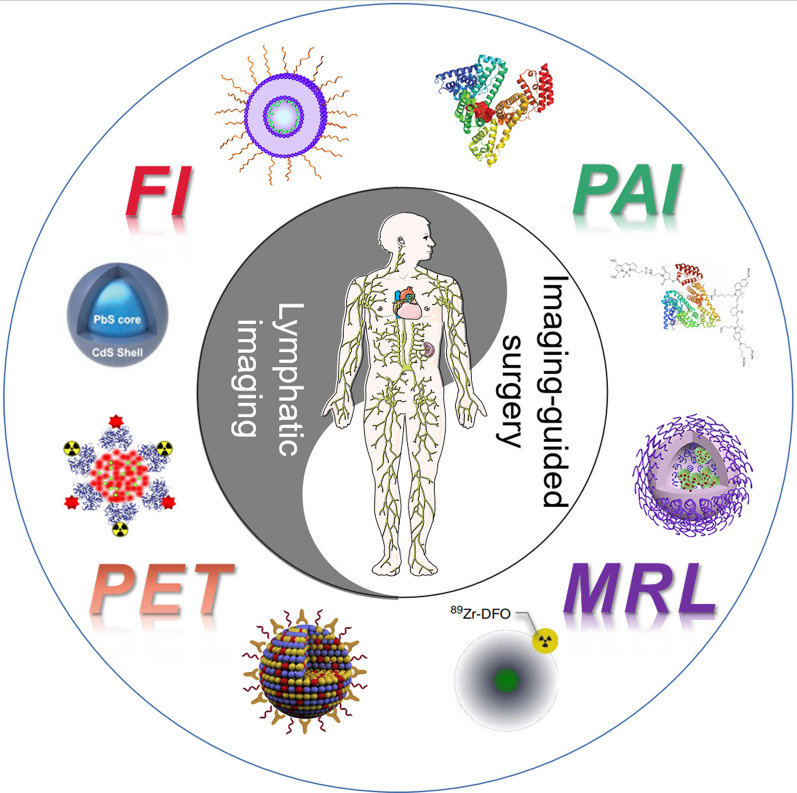

## Introduction

Blood and lymphatic vessel networks are two central components of cardiovascular system in vertebrate, playing a pivotal role in body homeostasis maintenance and involving in the development of multiple diseases [1]. Compared with blood circulation system, the research on lymphatic system has been neglected for a long time. The cognitive functions of lymphatic system mainly include the following aspects: (1) maintenance of tissue fluid homeostasis, (2) uptake of dietary lipids, (3) host defense and immunity, and (4) protein transportation. Increasing evidences have revealed that the lymphatic system is closely interrelated with cardiovascular disease, inflammation, cancer and obesity, which are the four major challenges to healthcare in the coming decades [2, 3].

As a significant transportation route for various soluble antigens and activated antigen-presenting cells from peripheral tissues to the draining lymph nodes, the immune cells in the lymphatic system undertake the important immunosurveillance functions [4]. Lymphatic endothelial cells (LECs) determine the adaptive immune response by mediating immune cell trafficking, promoting T cell tolerance and activation [5, 6]. The lymphatic vessels lined with LECs play an active role in formation of innate and adaptive immunity, and a suitable microenvironment is established by downstream lymph nodes as the confluence of migratory immune cells [7]. Once the anatomical association and functional coupling between the lymphoid organs are disrupted, pathogens bypass the tight surveillance of lymphatic system and access to the circulation, thereby eliciting illness [8]. Therefore, an intact lymphatic system is essential for maintaining host immunity and probing the pathogenesis of multiple diseases. Furthermore, to gain more insight into the kinetics and functions of lymphoid tissues and immune cells, the strategies of lymphatic imaging at different levels are desired, including whole-body imaging, intravital organ/cell imaging and ex vivo tissue/cell imaging [9].

The lymphatic system is characterized by slow unidirectional flow, colorless transparency and minor diameter, which make it difficult to be recognized even under microscopy [10]. Therefore, interstitial or subcutaneous injection of contrast agents are destined to be the most appropriate approach to observe the lymphatic system. However, the shortcomings of individual imaging modalities existing in current clinical applications hamper the depiction of intact lymphatic system including poor spatiotemporal resolution, limited depth penetration, large-scale equipment and radiation exposure [11]. Despite substantial efforts have been made to develop clinically available nanoformulations for lymphatic imaging, the progress in this filed is still unsatisfactory, promoting scientists to develop novel technologies for clinical lymphatic imaging.

Nanomaterials exhibit unparalleled advantages in the field of theranostic bioapplications benefiting from their excellent properties, including tunable structures, facile modifications, size effect and sensitive stimuli-responsiveness [12–15]. The improvement of biocompatibility, enhancement of resolution, and the relief of drug-associated side effects provide abundant possibilities for the treatments of a variety of diseases, thus inspiring the clinical translation [16]. Crucially, nanoscale formulations possess the native capability of passive targeting, which is ideally suitable for lymphatic imaging. Various studies on theranostic nanomaterials are currently in full swing worldwide, especially in the area of tumor and correlation sentinel lymph nodes (SLNs) imaging [17–24].

**Fig. 1:**
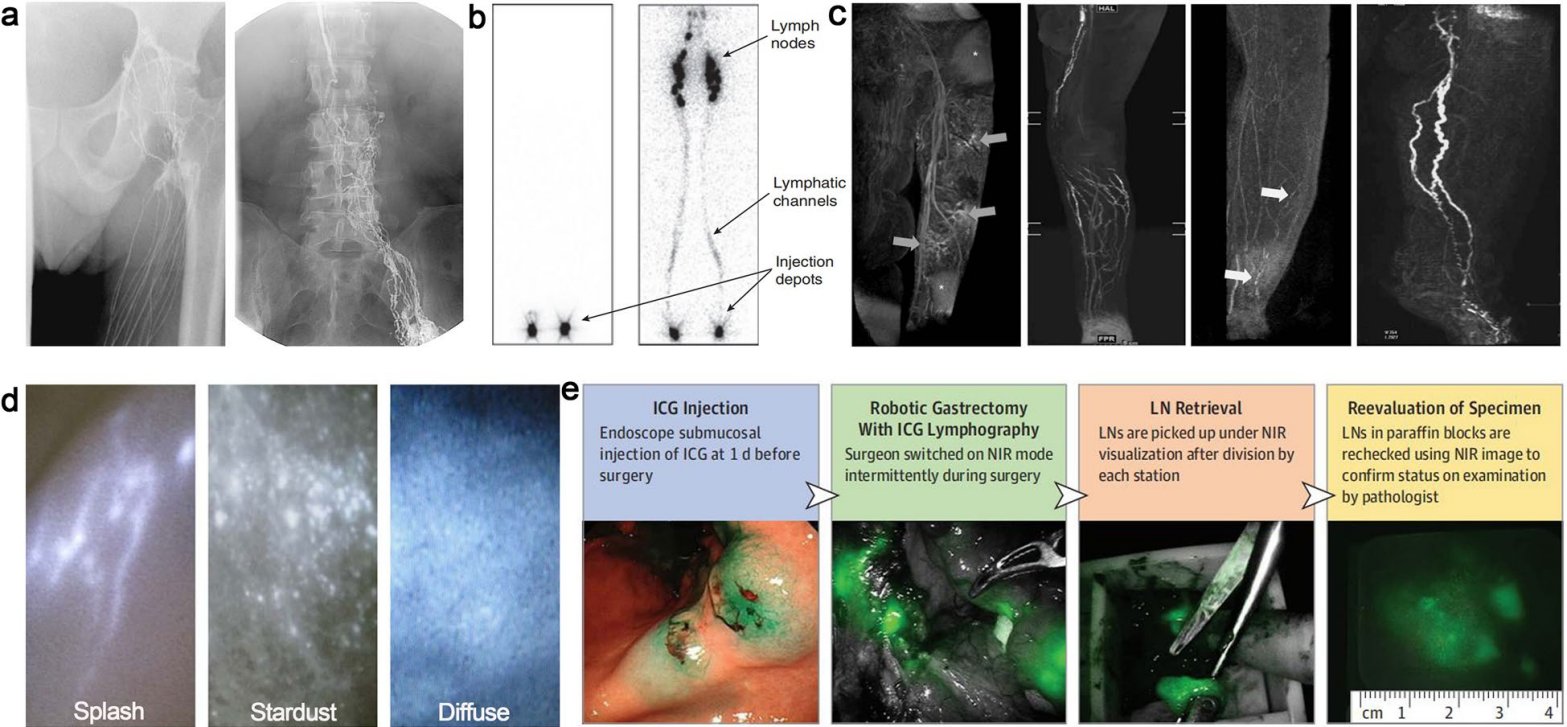
Current clinical imaging modalities of lymphatic system. **a** Typical radiographic image of the inguinal region following iodide angiography. Reproduced with permission [25]. Copyright 2019, Lippincott Williams and Wilkins. **b** Systemic lymphoscintigram after subcutaneous injection of radioactive tracers in the fee. Reprint with permission from [26]. Copyright 2010, Wiley–VCH Verlag GmbH & Co. KGaA, Weinheim. **c** Different MRL patterns of lymphatic drainage in lymphoedematous limbs. Reproduced with permission from [27]. Copyright 2015, Wiley–VCH Verlag GmbH & Co. KGaA, Weinheim. **d** Characteristic of indocyanine green (ICG) lymphography in different lymphedema stages, Reproduced with permission from [28]. Copyright 2014, SAGE Publications Inc. **e** Procedure to defining fluorescent LNs in robotic gastrectomy. Reproduced with permission from [29]. Copyright 2018, American Medical Association

**Scheme 1:**
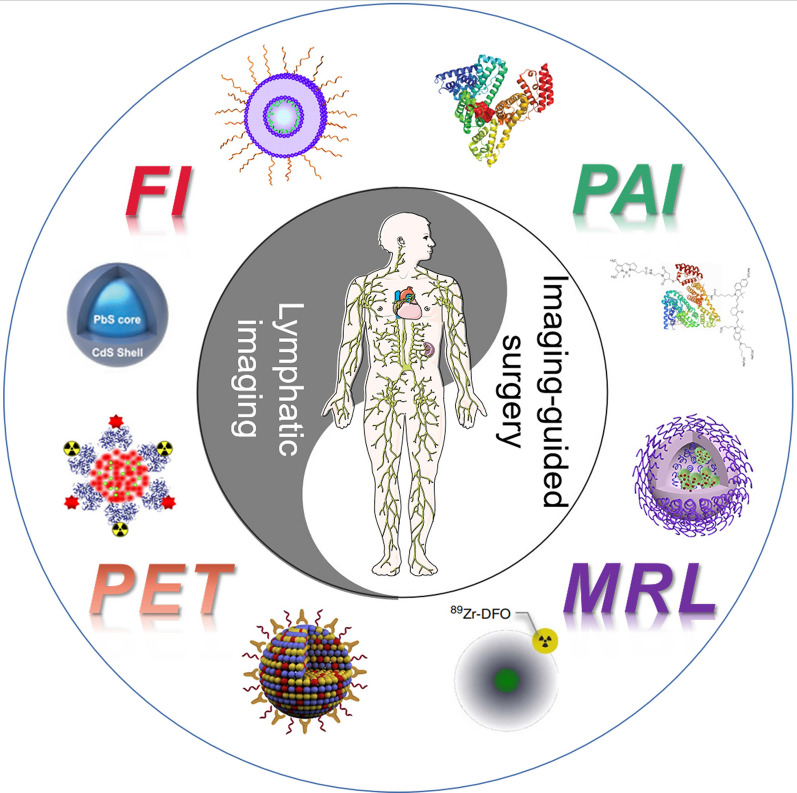
The application of nanotechnology in lymphatic imaging and imaging-guided surgery

In this review, we summarize the established approaches of lymphatic imaging used in clinic and introduce their strengths and weaknesses (Fig. 1 and Table 1). Several impact factors on the design of theranostic nanomaterials for lymphatic imaging are discussed. Subsequently, we highlight the recent advances in the field of preclinical lymphatic imaging, especially about the application of theranostic nanomaterials. We focus on the utilizations of nanomaterial to heighten the lymphatic image resolution, which improve the diagnostic accuracy and therapeutic efficacy (Scheme 1). We believe that this review will arouse extensive attentions and inspire more distinctive studies on the related research fields.

**Table 1:** Current clinical application of lymphatic resolution

Technique	Typical Tracers	Depth	Resolution	Advantages	Limitations	Applications	References
X-ray lymphography	Lipiodol	No limit	~ 1 mm	Deep tissue penetration	Invasiveness, Time consuming Radiation exposure	Central and collecting lymphatic imaging	[25, 30–33]
Lymphoscintigraphy/SPECT	^99m^Tc-coupled radioactive probes	No limit	1–1.5 cm	Deep tissue penetration, high sensitively	Exposure to ionizing radiation Planar image Poor spatiotemporal resolution	Visualization of collecting lymphatic vessels and dermal backflow, Quantitative assessment of lymphatic function, SLN mapping	[26, 34–38]
MR lymphography	Gd-based tracers or SPIO	No limit	0.5–2 mm	High imaging depth, 3D imaging can be realized without radiation	Low lymphatic specificity of clinically approved contrast, Venous signal interference, High cost	Collecting lymphangiography, Functional (dynamic contrast-enhanced MRL) and morphological evaluation of lymphatic vessels, SLN mapping	[27, 39–43]
Fluorescence imaging	Mainly ICG	1.5–2.0 cm	In the μm range (Depending on the instrument and depth)	Simple operation, no ionizing radiation, high temporal and spatial resolution, low costs	Limited depth of imaging, serious self-aggregation, lack of better clinically-approved tracers	Precise imaging of peripheral lymphatic vessels, Visualization of dermal backflow and quantitative assessment of lymphatic function, SLN mapping	[28, 29, 44–47]

## Lymphatic imaging strategies

Nanoparticles used for lymphatic imaging usually referred to the size of 5–100 nm, locating in the “just-right” region for lymphatic drainage. The subtle size endows nanoparticles with prolonged retention in circulatory system, enhancing imaging resolution and pharmaceutical effect, rather than being trapped in the capillaries of the organs. Actually, both the characteristics of nanomaterials and lymphatic structure have great influence on the imaging results. In this section, we mainly introduce the special characteristics of lymphatic structure and the strategies for the design and delivery of nanoparticles.

### Lymphatic structure

**Fig. 2:**
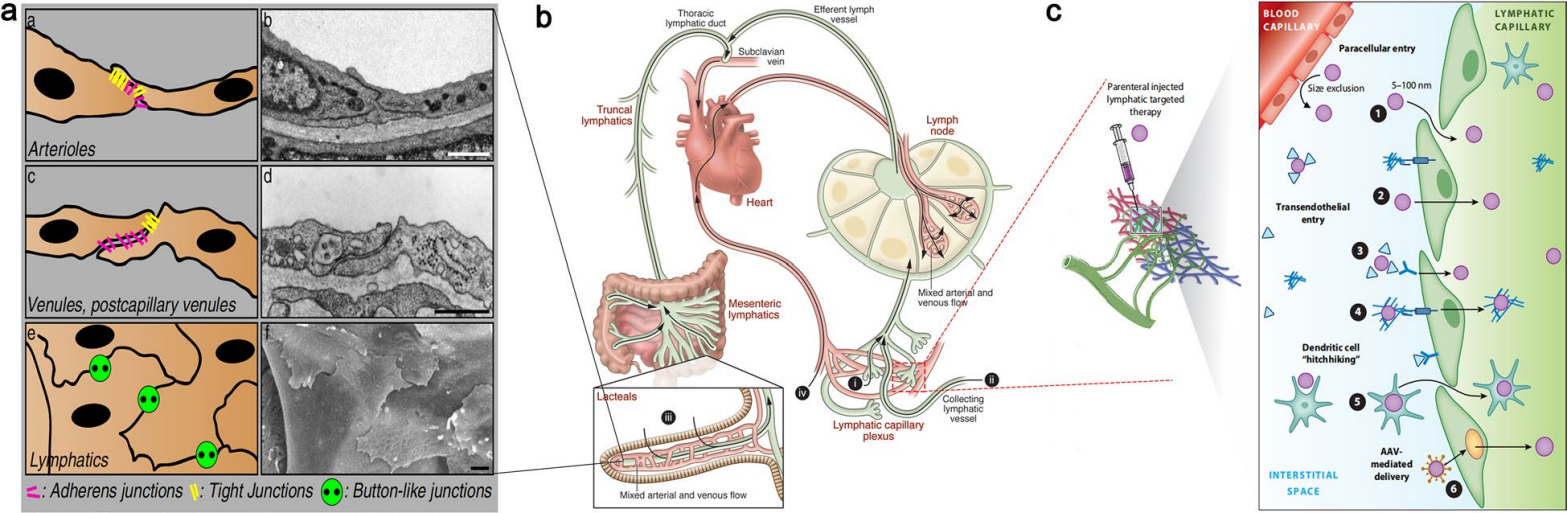
**a** Three types of endothelial junctions (left) and the corresponding electron micrographs (right). Reprint with permission from [50]. Copyright 2009, Springer-Verlag GmbH Germany. **b** Organization chart of whole-body lymphatic system and the routes of contrast agent administration. (i) Intradermal/interstitial administration or indirect lymphography. (ii) Intra-lymphatic administration or direct lymphography. (iii) Oral gavage of hydrophobic lipids results in uptake through the lacteals into the mesenteric lymphatics. (iv) Intravenous administration of lymphatic contrast. Reproduced with permission from [53]. Copyright 2014, American Society for Clinical Investigation. **c** Modes of drug or contrast entry into lymphatics from the interstitium. Lymphatic-targeted drugs delivered to the interstitial space are passively or actively shuttled into lymphatic capillaries dependent upon the chemical and physical properties of the drug or drug delivery system. Reproduced with permission from [63]. Copyright 2021, Annual Reviews

The lymphatic capillaries are ducts with a closed blind end, which consist of single-layer LECs and incomplete basement membrane [48]. Unlike vascular endothelial cells, LECs adopt a tile-like or fish-scale structure, in which the endothelial cells overlap the edges of adjacent ones (Fig. 2a) [49, 50]. Such a distinctive arrangement allows macromolecular particles (such as proteins, lipids) and immune cells suspending in the interstitial enter lymphatic capillaries through the endothelial gap without backflow [51, 52]. When the extracellular matrix (ECM) is swollen (such as subcutaneous injection of nanomaterials), the gap of LECs is opened to ~ 100 nm by the interaction between anchoring filaments and collagen fibres, and subsequently the nanomaterials enter lymphatic capillaries. The nanomaterials migrate to the primary lymph nodes followed by entering the next lymph nodes or thoracic duct and finally flow into the blood circulation (Fig. 2b) [5, 53]. Thus, the nanomaterials can achieve their specific theranostic functions following the lymphatic recycle. About 80% of the lymph comes from liver and intestinal lymphatics which are rich in proteins and lipids, the lymphatic function of liver and intestine can provide more insights into the design of lymphatic theranostic nanomaterials [54].

### Tracer design and delivery

It is widely recognized that the size of the nanomaterials determines its selective entrance in blood or lymphatic vessels. Typically, nanoparticles 5 to 100 nm in diameter are suitable for lymphatic drainage, depend on the maximum gap (ca.120 nm) between the endothelial cells of lymphatic capillary [55, 56]. However, the transport mechanism of macromolecules passing through LECs is not fully understood [57–59]. Different from the previous wisdom, recent studies have shown that a few pathways play significant roles in the lymphatic transport of macromolecules [60]. Consistent with this concept, the clearance of hyaluronic acid in interstitium is mostly related to its interaction with LYVE-1 that specifically expression on LECs [61]. Schubert et al.proposed that albumin was transported across vascular endothelium by gp60 and caveolae transporters [62]. Although the mechanism of LEC surface receptors facilitate the entrance of macromolecules into lymphatic vessels remains confused, the receptor-mediated ingestion mechanism provides a potential avenue for precise lymphatic imaging and theranostic applications (Fig. 2c) [63].

Apart from the size, surface charge also has a great influence on the delivery efficiency. In this regard, it is difficult to reach consensus conclusion about the influence of surface charge in lymphatic imaging, because both negatively and positively charged nanoparticles showing efficient lymphatic drainage and retention [64–69]. As the major components of ECM, hyaluronic acid (HA) with negative charge plays a positive role in the clearance of contrast agents. Negatively charged nanoparticles are preferred for lymphatic imaging due to the weaker interactions with HA and specially stress-induced transport mechanism [70, 71]. However, the positively charged NPs exhibit higher endocytosis compared to negatively or natural ones owing to the negative surface charge of the cell membrane. Ma et al*.* demonstrated that the same size of NPs but different zeta potentials (+ 39.25, + 0.51 and − 45.84 mV respectively) showed different cellular uptake, the cationic NPs exhibited higher cellular uptake than the other two in eight different cell lines consisting of epithelial cells, fibroblastic cells, carcinoma and megakaryoblastic cells [72]. Helle et al. also confirmed that when the negative charges were hidden by PEG shell, silica nanoparticles-COOH_core_ (35–40 nm) seemed more appropriate in mapping lymph nodes after subcutaneous injection [68]. Interestingly, Huang et al*.* demonstrated that negatively-charged NPs had higher accumulation than positively charged NPs in lymph nodes after *i.v.* injection, whereas positively charged NPs exhibited higher gene expression level [73]. Taking all these factors into consideration, negatively charged nanomaterials smaller than 100 nm can be transported from interstitial into lymphatic vessels, while the positively charged nanoparticles are possibly brilliant candidates for activating the immune response or exerting the pharmacodynamic effect by efficient endocytosis.

Besides the factors mentioned above, the sites of subcutaneous injection also play the important role in lymphatic imaging. Kagan et al*.* assessed the relationship between lymphatic absorption and the injection site by injecting three distinct macromolecules into lateral thigh, including bovine insulin (5.6 kDa), recombinant human α-erythropoietin (30.4 kDa) and bovine albumin (66 kDa)*.* It was found that the accumulation of three proteins with different molecular weights was almost barely detected (< 3%) in thoracic duct. The author attributed this phenomenon to the difference of anatomical structure of the injection site, which has extensive diffusion space and could not supply effective tissue hydrostatic pressure [74]. Similar conclusion was drawn in a liposome biodistribution study by Oussoren et al.[75]. These findings warrant more attention since the preclinical evaluation of nanoformulations is becoming more widespread, including vaccines, immunization and lymphatic imaging.

## State-of-the-art of nanotechnology in preclinical lymphatic imaging

To date, a large number of theranostic nanomaterials have been reported for preclinical lymphatic imaging studies [76–80]. The following sections highlight recent advances in lymphatic imaging using sophisticated nanoformulations, intraoperative navigation and sentinel lymph node biopsy in detail.

### Radionuclide imaging

**Fig. 3:**
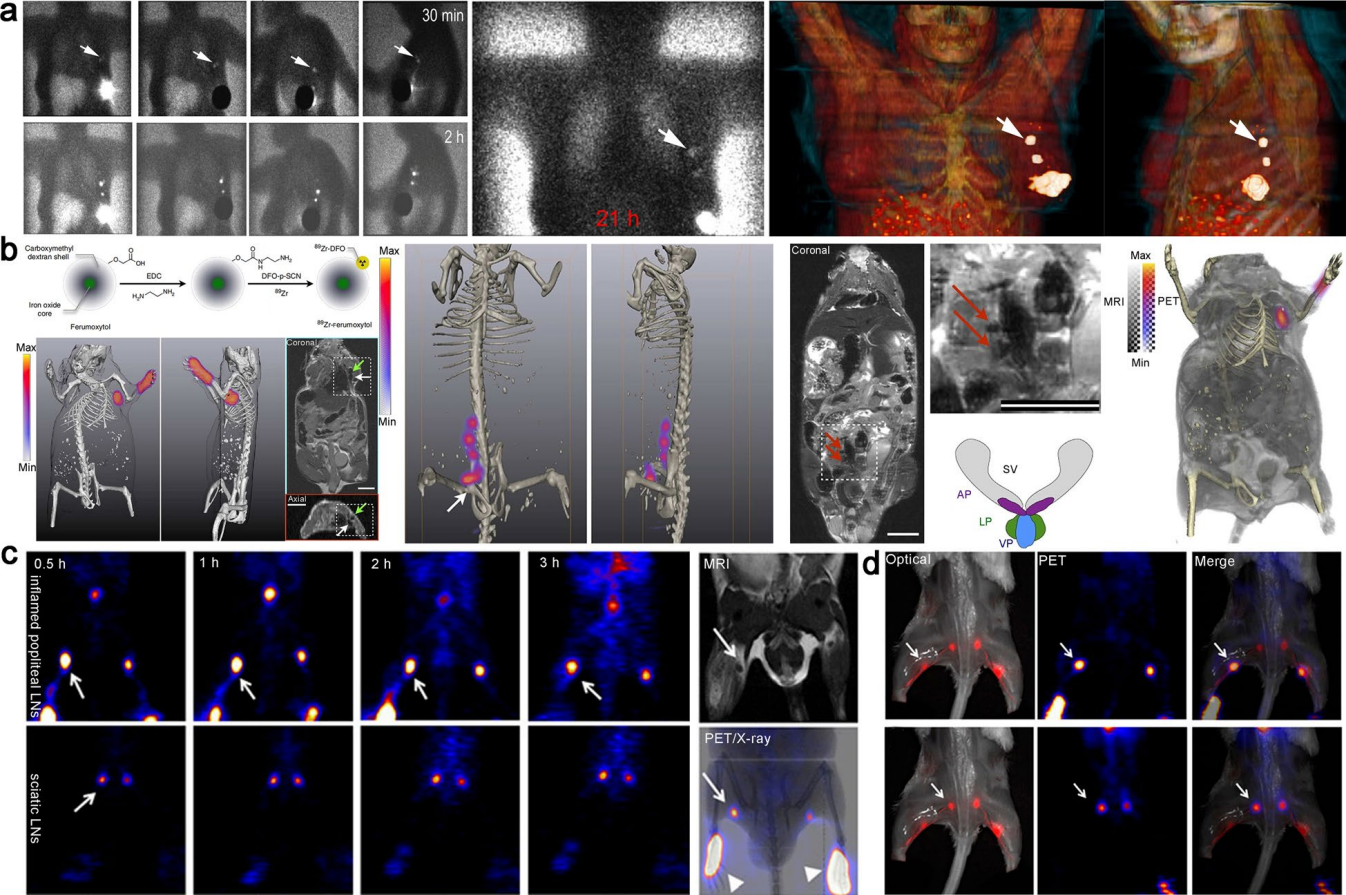
**a** The images obtained by intratumoral injection of ^99m^Tc-Tilmanocept contrast agent in a patient with breast cancer. Two well-depicted SLNs were clearly seen in all sets of images (white arrow). Reproduced with permission from [85]. Copyright 2021, Society of Nuclear Medicine and Molecular Imaging. **b** Schematic of ^89^Zr-ferumoxytol and multimodal visualization of lymph nodes (axillary and somatic). Reproduced with permission from [87]. **c** The reconstructed coronal PET images of inflamed popliteal (Upper) and sciatic (Lower) LNs in the secondary hind limb inflammation model. **d** Complementary of optical (left) and PET (middle) images of enhancement popliteal and sciatic LNs, indicated by a white arrow. Reproduced with permission from [88]. Copyright 2014, Society of Nuclear Medicine and Molecular Imaging

Lymphoscintigraphy (LSG) is recommended as the test of choice for lymphedema patients, with a Grade 1 recommendation and B level of evidence by the American Venous Forum, which is also widely used for seeking SLNs of tumor [81, 82]. The capability of fast clearance from injection site, accurate accumulation and high retention are essential for SLNs imaging. In order to fulfill these requirements, ^99m^Tc-tilmanocept, a novel CD206-targeting lymphatic imaging agent was approved by FDA in 2014. It can be specifically and rapidly taken up by lymphatic vessels owing to its relatively small size (about 7 nm), and allowing accurate identification and retention in SLNs by mannose-mediated T cells endocytosis (Fig. 3a) [83–85].

On the basis of planar lymphoscintigraphy, PET/CT or SPECT/CT provides complementary 3D images. From the efficacy and biosafety perspective, Wang et al*.* reported a ^99m^Tc-labeled biomineralization nanoparticle for multimodal imaging of lymphatic drainage and network in real-time using SPECT/MRI and NIR fluorescence. The gadolinium oxide nanoparticles were synthesized by the biomineralization approach from Gd and ovalbumin, in which the radioactive tracer and cypate was conjugated to ovalbumin scaffold. The corresponding MRI/NIR/SPECT nanoparticles (^99m^Tc-Gd@OVA-Cy) with the appropriate size exhibited high resolution and sensitivity lymphatic imaging [86]. Compared with SPECT, PET lymphography provides higher sensitivity and resolution in imaging of lymphatic system and SLNs by intradermal injection of radioisotope-labeled tracers, such as ^18^F-FDG. However, ^18^ F-FDG, a small molecule, is rapidly eliminated by blood circulation after local injection, which in turn hamper lymphatic system imaging. Thorek et al. introduced a multimodal nanoparticle,^89^Zr-ferumoxytol (ca. 17–35 nm), to precisely detect LNs on the basis of PET/MRI imaging. When the tracer was administered in the forepaw (superficial tissue) and prostate (deep), lymph nodes were clearly observed by PET/MRI modality using 3D visualization and reconstruction with a lower specific activity range of 0.1–0.5 mCi mg^–1^ Fe (Fig. 3b) [87]. Chen et al*.* synthesised a nanomaterial (^18^F-AIF-NEB) by combining ^18^F aluminum fluoride-labeled NOTA conjugated truncated Evans blue (EB) for lymphatic imaging. ^18^F-AIF-NEB rapidly formed stable complex with endogenous albumin when it was injected into the interstitial space. The formed complex specifically drained into the lymphatic system, allowing multimodal PET/Optical imaging of the lymphatic system. This “hitchhiking” strategy not only greatly improves the fluorescent quantum yield of fluorescent dyes, but also reflects the behavior of endogenous proteins in the body through radioactive signals, avoiding the use of colloids, polymers and nanoparticles. It provides new insights for future design of contrast agents with brilliant safety, which exhibits great potentials in preoperative assessment and intraoperative navigation of lymphatic diseases (Fig. 3c) [88].

**Fig. 4:**
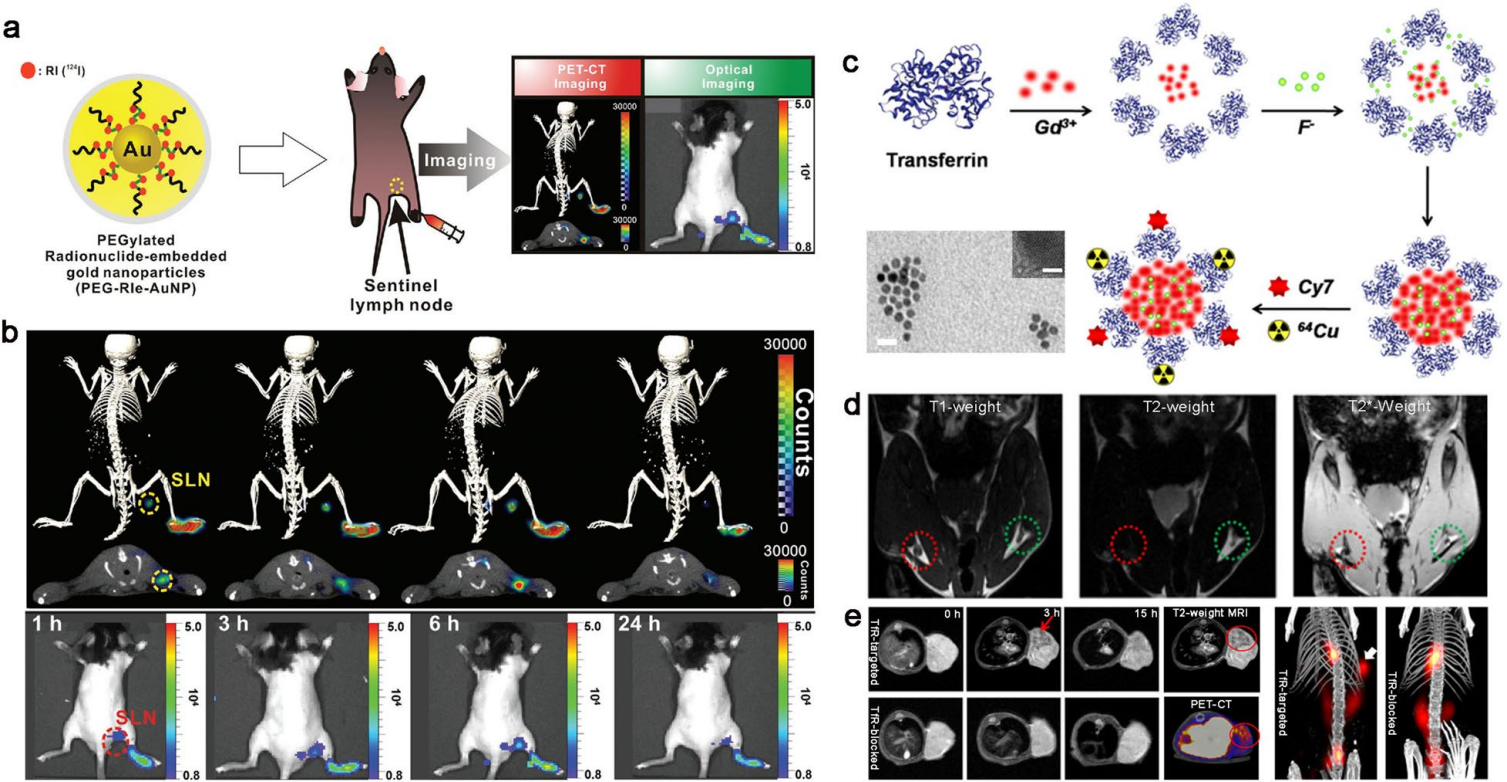
**a** Schematic illustration of PEG-RIe-AuNPs and multiple images of sentinel lymph nodes in vivo. **b** PET/CLI signal of PEG-RIe-AuNPs accumulate in sentinel lymph nodes over time. Detailed: (upper) 3D-PET/CT images, (middle) PET/CT images of cross-sectional, (bottom) CLI images. Reprinted with permission [89]. Copyright 2016, Wiley–VCH Verlag GmbH & Co. KGaA, Weinheim. **c** Self-assembly and modification of transferrin encapsulated GdF_3_ nanoparticles and TEM images. Scale bars are 100 nm (TEM) and 1 nm (HRTEM). **d** T_1_ and T_2_-weighted MRI images of SLNs in healthy mouse after injection of GdF_3_@Tf NPs 3 h. **e** Multimodal imaging of colorectal tumor bearing-mice at different time points after intravenous injection of ^64^Cu-GdF_3_@Tf-Cy7 NPs. Reproduced from [90]. Copyright 2020, American Chemical Society

Although there are various types of radiolabeled metal nanomaterials holding great application potentials in bioimaging, the conventional manner of electrostatic attraction or simple chelation can easily lead to radioisotope translocation to proteins, thus subsequently resulting in image misreading. A novel nuclear theranostic platform, ^124^I embedded gold (Au) nanoparticles (RIe-AuNPs), was developed by Jeon and co-workers via straightforward DNA-based radiolabeling chemistry and additional hybridization strategy. Combining PET and Cerenkov luminescence imaging (PET/CLI), PEGylated RIe-AuNPs enabled the sensitive and stable mapping of sentinel lymph nodes in vivo, and the injection dose and radioactivity (10 × 10^−12^ M/1.295 MBq) were extremely low. More importantly, the building blocks utilized were commercially available, favorable for the clinical translations (Fig. 4a, b) [89]. Recently, Gao et al. used biomineralization method to develop a transferrin-encapsulated GdF_3_ nanoparticles (GdF_3_@Tf NPs) with size of 40–50 nm. Cy7 and ^64^Cu were further employed to modify GdF_3_@Tf NPs to endow the nanomaterial with NIR and PET imaging capability (^64^Cu-GdF_3_@Tf-Cy7 NPs). The GdF_3_@Tf NPs exhibit high T_2_-weighted enhancement compared with most T_1_-weighted rolled contrast agents. The nanoparticles realized multi-functional imaging (PET, CT, MRI and fluorescence) with excellent tumor accumulation specificity by taking advantage of transferrin-mediated targeting delivery, exhibit great potential in clinical translation (Fig. 4c–e) [90].

**Fig. 5:**
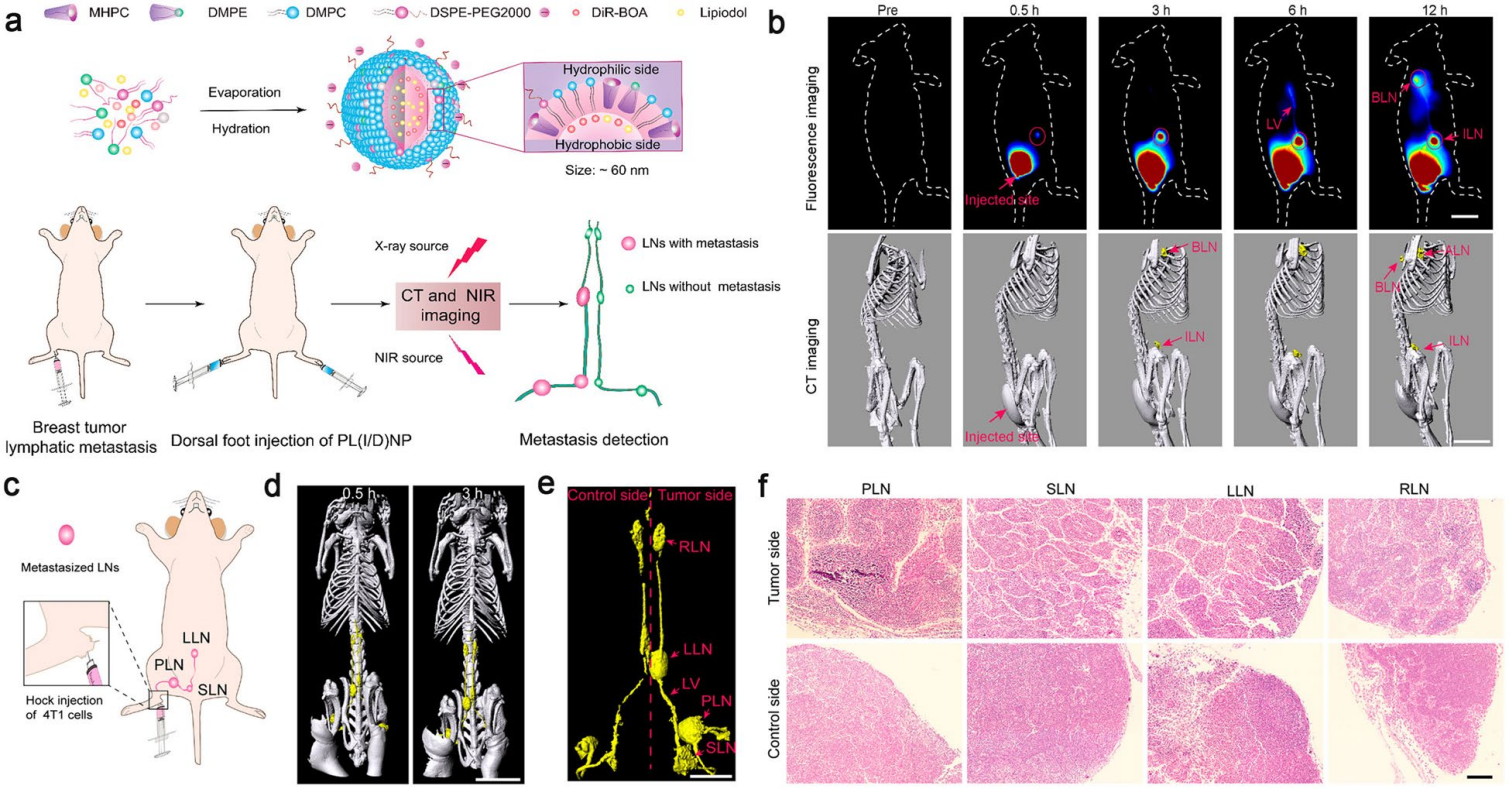
**a** The illustration of the synthesis process of PL(I/D)NPs and the metastasis LN detection by CT and NIR imaging in vivo after dorsal foot injection of PL(I/D)NPs. **b** Representative fluorescence and 3D reconstructed CT images of superficial lymphatics with PL(I/D)NPs in vivo after tail subcutaneous injection. **c** Schematic illustration of metastasis LNs after hock injection. **d** The CT reconstruction images of mice lymphatic system. **e** Representative images of lymphatic architecture extracted from d. **f** H&E staining of LNs corresponding to e. Reproduced from [91]. Copyright 2020, American Chemical Society

Lipiodol once played a significant role in the imaging of lymphatic system at early stage. This hydrophobic opaque agent can remain in the lymph vessels and indirectly show the profile of lymph vessels and nodes. However, this strategy is rarely used in clinic because the lipiodol needs to be injected into lymph vessels directly, which is technically difficult to operate and increase the risk of pulmonary embolization. Zhang et al*.* developed a phospholipid nanoparticle (PL(I/D)NPs, ca. 60 nm) core-loaded with lipiodol (I) and a near-infrared dye (DiR-BOA) to trace the lymphatic system. Scavenger receptor class B member 1 (SR-B1), a key surface receptor for anionic phospholipids, is highly expressed in the endothelial cell of lymphatic vessels, which facilitates the special translation of phospholipid nanoparticle into the lymph vessels. PL(I/D)NPs provided a long-distance map of the lymphatic system, from the subcutaneous injection site in feet to the thoracic ducts with an entire length about 68 mm. Meanwhile, the metastatic LNs were identified by measuring individual volume and the separation distance along the lymph vessels. This noninvasive identification strategy integrates active and passive targeting capabilities will bring new enlightenment to the clinical whole-body lymphatic system imaging (Fig. 5) [91].

### Magnetic resonance lymphography

**Fig. 6:**
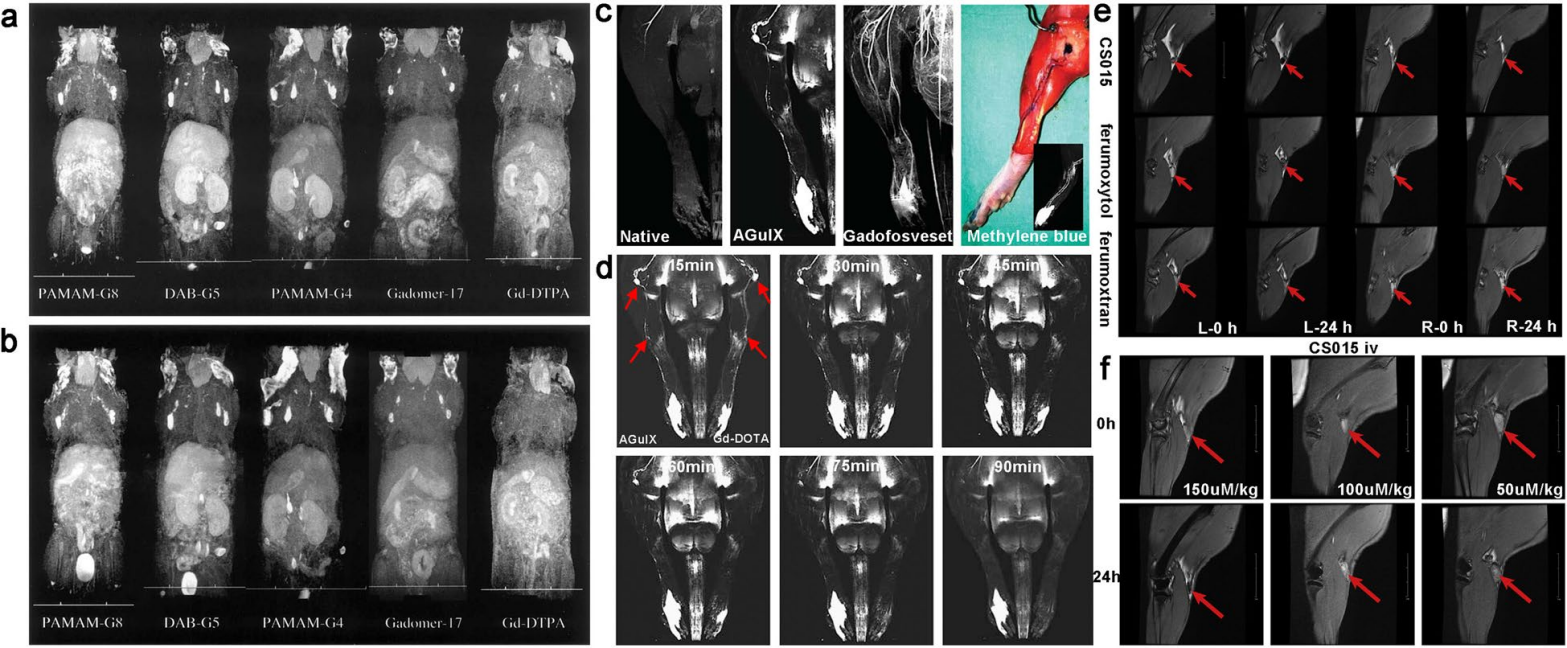
**a**, **b** The whole-body dynamic 3D-micro-magnetic resonance lymphangiography of mice injected with PAMAM-G8, DAB-G5, PAMAM-G4, Gadomer-17 and Gd-DTPA into foot gap. The MR images acquired at 10 and 45 min post-injection are shown, with permission from [95]. Copyright 2003, Wiley–VCH Verlag GmbH & Co. KGaA, Weinheim. **c** The magnetic resonance imaging and magnetic resonance angiography in rats. First two pictures: 3D-TOF-GRE hindlimb scan before (Native) and after (AGulX) intradermal AGuIX injection. Third: 3D-GRE angiography with intravenous Gadofosveset after AGuIX administrated 2 h. Last: Anatomical dissection after methylene blue injection. **d** Representative MRL images with AGuIX and Gd-DOTA in different time. The arrowhead shows the lymph nodes and collecting lymphatic vessels. Modified from [97]. Copyright 2017, Wolters Kluwer Health. **e** Comparison of CS015 and two commercially SPIOs for enhanced MR angiography with subcutaneous injection. **f** MR images of lymph nodes before and 24 h after intravenous injection of CS015. Reprinted with permission [103]. Copyright 2020, Elsevier

Magnetic resonance imaging (MRI) exhibits a growth advantage in the diagnosis of lymphatic disorders due to its excellent performance in imaging of soft tissue and slow fluid [92]. Magnetic resonance lymphography (MRL) allows fine visualization of the deep lymphatic system and regional lymph nodes with higher resolution. In recent years, it is notably favored by clinicians as the constant development of macromolecule contrast agents (mCAs). The mostly used MRI mCAs are T_1_-weighted paramagnetic transition metal ion chelates gadolinium (Gd) chelates as examples, and T_2_-weighted superparamagnetic iron oxide nanoparticles (SPIO) [93]. The injection dose of gadolinium-based MRI mCAs is 100-fold lower than that of iodine atoms required for CT imaging. Ideal MRI contrast agents are desired to possess high efficiency and minimum side-effects, without the possibility to introduce harmful ingredients into the body [94]. Kobayashi et al*.* compared the imaging performances of three dendrimer-based contrast agents with different sizes, PAMAM-G8 (958 kDa), DAB-G5 (51 kDa) and PAMAM-G4 (58 kDa), aiming to analyzing the pharmacokinetic behavior of these mCAs. In vivo experiments revealed that hydrophilic PAMAM-G8 with larger size was more favorable for lymphatic vessel visualization, while the smaller hydrophobic DAB-G5 was more effective for lymph node imaging. The hydrophilic PAMAM-G4 was intermediate in character between PAMAM-G8 and DAB-G5, but exhibiting the lowest background signal in the liver. PAMAM-G4 was also regarded as the preferred choice for clinical application due to the fast clearance and low background signal (Fig. 6a, b) [95, 96]. In this study, the author used the size effect to ingeniously reduce the possible related nephrotoxicity. When PAMAM-G4 was transferred from the lymphatic system to the blood circulation, it should be rapidly excreted from the kidney without significant retention.

DOTA is given preference by virtue of its extremely high association constant. The better stability of thermodynamic and kinetic limit transmetallation with endogenous cations (e. g., Zn^2+^, Ca^2+^) and reduce the release of toxic Gd^3+^ ions. Additionally, nanoparticles around 3–10 nm can be selectively untaken by lymphatic system and easily excreted from kidney. Müller et al*.* evaluated the MRL performance of gadolinium (Gd)-based ultra-small nanoparticle AGuIX (3–4 nm) in healthy rats and chronic hindlimb lymphedema models. In the rat hindlimb lymphedema model, AGuIX enabled MRL of anatomical depiction with a higher-resolution (Fig. 6c, d) [97]. Compared with the non-operated hind limb, AGuIX MRL indicated that thicker lateral lymphatic draining and subcutaneous reflux have emerged at the distal end of the ligated lymphatic vessels after lymphovascular ligation six weeks. MRL showed a significant reduction in their collateral lymphatics 10 to 14 weeks post-operation, which was owing to the onset of the compensatory mechanisms in chronic lymphedema. In order to avoid the potential side effects caused by the release of Gd ions from DTPA over time, Yano used cyclic DOTA as an alternative chelator, which was employed to develop a new contrast agent using a carboxylated nanodiamond (CND) as a platform. Compared with Gd-DTPA-CND, the newly fabricated Gd-DOTA-CND contained fewer Gd elements, but exhibited stronger MRI signal in water and serum, showing promising potential in clinical MRL imaging [98].

Superparamagnetic iron oxide (SPIO) nanoparticles effectively migrate to lymph nodes after local administration due to phagocytosis by macrophages. The accumulation efficiency of SPIO in tumor metastatic lymph nodes is much lower than that in normal, so the heterogeneous signal enhancement can be used to identify whether enlarged lymph node is associated with tumor metastasis [99–101]. Turkbey et al*.* conducted a study on 49 patients suspected of lymph node metastases with prostate, bladder and kidney cancer. SPIO nanoparticles were intravenously injected, MRI scans of the region of interest (ROI) were performed at 24 and 48 h post injection. The specificity, sensitivity, negative predictive and positive predictive value were 64.4%, 98.0%, 93.5% and 86.0% respectively, which were independent on the lymph node size. The penetration process of SPIO particles into the lymphatic system was very slow after intravenous injection, requiring delayed imaging [102]. Recently, Gu et al. synthesized a nanoformulation (CS015, ca. 30 nm) with the capability of MRL at an ultra-low effective dose (0.075 μmol/kg). Surface modified PAA segments could effectively prevent the self-aggregation, boosting the absorption by lymphatic system. Compared with two commercially available SPIO nanoparticles ferumoxytol and ferumoxtran-10, CS015 with negative charge (− 44.5 ± 0.50 mV) exhibited excellent imaging resolution of popliteal lymph nodes. After intravenous injection of CS015 was mainly distributed in the liver, spleen and systemic lymph nodes, which is a good option for the imaging of systemic lymph nodes 24 h post intravenous injection (Fig. 6e–f) [103]. Though the iron oxide nanoparticles as a T_2_ MRI contrast agents have improved sensitivity of lymphatic imaging, the negative signal present challenges in issues imaging with intrinsically low MRI signals [104]. The magnetic particle imaging (MPI) emerges, which measures the change in electronic magnetization of iron and promises much higher sensitivity than MRI in vivo. Song et al. developed a sensitive and multimodal imaging tracer, carbon-coated FeCo nanoparticles about 10 nm. The MPI signal intensity was sixfold and 15-fold higher than the signals from two kinds of commercial superparamagnetic iron oxide tracers, VivoTrax and Feraheme. FeCo@C-PEG nanoparticles also had high optical absorbance in a broad NIR region (700–1200 nm) and exhibited great potential in lymphatic photoacoustic imaging [105]. The toxicity of metal-based NPs is always a concern, the reactive oxygen species (ROS) mediated cytotoxicity is considered as an important mechanism of renal toxicity induced by metal-based nanoparticles. An experiment was performed to study the effect of Fe_3_O_4_ NPs on the kidney, which indicated that the increased lipid peroxidation was significant only at the highest exposure dose (40 mg/kg) and the oxidative stress was mainly induced by the release of Fe from Fe NPs [106].

Several breakthroughs have achieved in MRL over the past years by fully exploiting the nanotechnology, there still exist several issues during clinical applications. For instance, it’s difficult to distinguish dilated lymphatic vessels from the concomitant venous. The main way to distinguish these two vessels in clinical is mainly based on the imaging features, such as ductal diameter, morphology, and bead-like changes. Actually, adding an MR venogram after the depiction of the lymphatic system is also an acceptable approach [27]. Another alternative solution is performing a 3D steady-state free precession (SSFP) balanced electrocardiography-(ECG) triggered sequence with spectral fat saturation instead of a heavily T_2_-weighted sequence before 3D gradient-echo T_1_-weighted MRL. In this way, the severity and distribution of lymphedema are directly visualized and the veins are displayed more clearly [107]. It is essential to assess the location and activity of lymphatic vessels using MRL in preoperative of lymphatic venous anastomosis, however the limited information obtained by MRL emphasizes the importance of combining with other imaging methods, such as fluorescence imaging (ICG) [108, 109].

### Fluorescence imaging

**Fig. 7:**
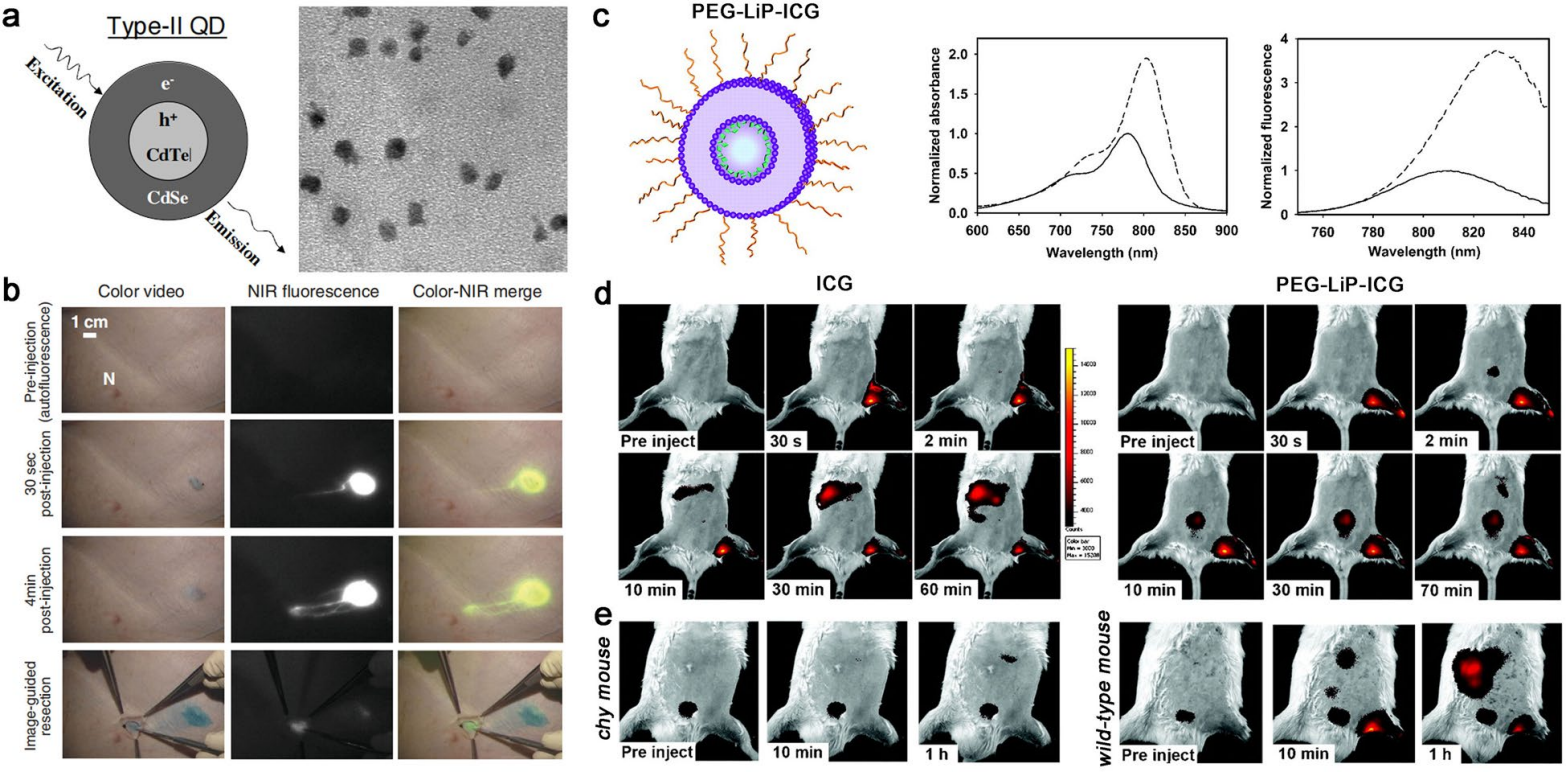
**a** Structure of conventional type-II QDs and TEM. **b** Images of the surgical field in a pig injected intradermally at the right groin with 400 pmol of NIR QDs. Reproduced with permission from [118]. Copyright 2004, Nature publishing group. **c** Structure of PEG-Lip-ICG and ICG was well dispersed, the UV–vis absorption spectra and OP fluorescence spectra are shown in the later pictures. **d** NIRF images from animals before and after intradermal injection of ICG and PEG-Lip-ICG in the left rear paw with series of time. **e** Representative images of Chy mouse (left), a model of lymphatic dysfunction and Wild-type littermate mice (right) after intradermal injection of PEG-Lip-ICG. Reprinted with permission [123]. Copyright 2010, American association cancer research

Fluorescence lymphatic imaging is coming into focus in recent years because of its excellent resolution, ease of usage, non-invasive and negligible radiation exposure [110–113]. Various fluorescent dyes have been developed for imaging of superficial lymphatic system, especially the fluorophore with the emission at the second near-infrared (NIR-II) window [114–117]. Early work mainly focuses on the use of near-infrared quantum dots (NIR QDs) for SLN detection. Type II QDs are widely used in pre-clinical research by virtue of their near-infrared emission, efficient light absorption and excellent photostability [117]. Frangioni et al*.* developed a new sort of type II QDs coating by oligomeric phosphine to render them soluble and stable in serum. Only 400 pmol type II QDs permit SLNs to be visualized feasibly in real-time using excitation fluorescence rates of 5 mW/cm^2^. The brilliant fluorescence behavior allows the type II QDs for imaging-guided operation of sentinel lymph node resection in large animals (pigs, closer to human size). It deepened the insight into operation filed, shortened the procedure time, narrowed surgical incision and improved the surgical efficiency. However, considerable challenges of QDs still remain due to the particle retention in the reticuloendothelial system of mice even after two years (Fig. 7a, b) [118].

Compared with QDs, ICG-encapsulated nanoplatforms have been extensively investigated owing to their excellent biosafety and ease of clinical translations. ICG is the only near-infrared fluorescent dye approved by FDA for clinical use, while its clinical applications are greatly hampered by the intrinsic defects, such as poor photo-stability, easy aggregation and quick clearance. In order to overcome the problems mentioned above during clinical applications of ICG, a majority of nanoformulations have been developed [120–124]. Proulx et al. introduced a PEG-modified liposome encapsulating ICG (PEG-Lip-ICG) for quantitative description of the dynamic flow in the lymphatic system. In the PEG-Lip-ICG nanoplatform (ca. 60 nm), ICG was homogeneously dispersed in the bilayer membrane, leading to a significant enhancement of fluorescence and avoiding self-aggregation in the interstitial matrix. PEGylation not only improved the solubility and stability in physiological environment, but also avoided the phagocytosis by the reticuloendothelium. More interestingly, a 22 nm red shift in fluorescence spectrum was observed after the formation of PEG-Lip-ICG, facilitating deeper tissue imaging. Compared with wide-type mouse, PEG-Lip-ICG showed significant lag lymphatic drainage and slow clearance at the injection site in a genetic mouse model of lymphatic dysfunction (Chy mouse) (Fig. 7c–e). The stability of PEG-Lip-ICG was satisfactory, less than 10% of ICG leaked from the nanoformulation within 3 h in the presence of low concentration serum, while rapidly dissociated in high concentration serum. This is conducive to the disintegration of nanoparticles into monomers for rapid metabolism after being transported to the blood system, which is beneficial for further design of degradable theranostic nanomaterials [123].

**Fig. 8:**
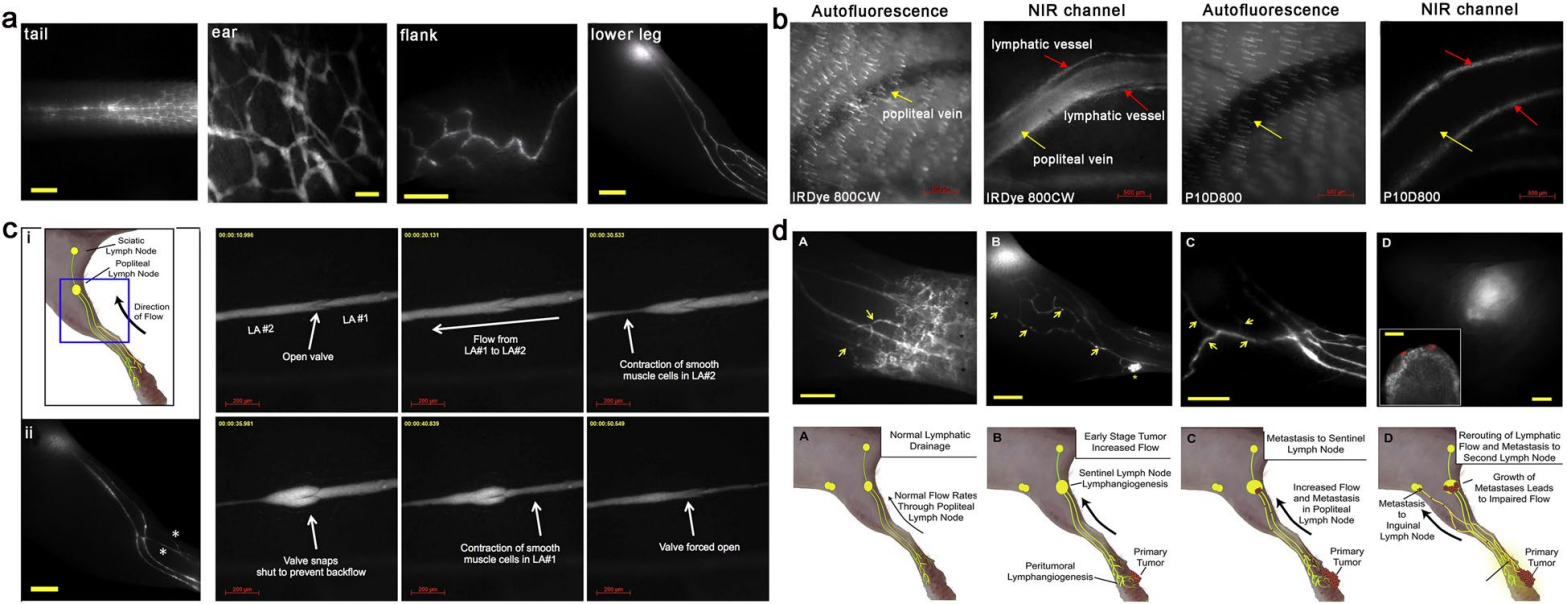
**a** Representative imaging of lateral side of lymphatic vessels in the tail, ear, flank and lower leg after intradermal injection of P20D680. **b** Specificity of tracer uptake by lymphatic vessels and popliteal vein after intradermal injection into hind paw with IRDye 800CW (ca.2 nm) and P10D800 (ca.7 nm). **c** The schematic diagram of lymphatic vessels network in mouse hind limbs (i) and the visualization of high uptake of P20D680 after intradermal injection into hind paw (ii). (iii) The valve function of collecting lymphatic vessels was also visualized in real time by P40D800. **d** Cartoon of lymphatic flow and rerouting of lymphatic flow after tumor spread from footpad tumors. Reproduced with permission from [124]. Copyright 2013, Elsevier

Although the combination of liposome and ICG increased the brightness, stability, and specificity to lymphatic system. The relatively large size, prolonged circulation and migration rates act as barriers to longitudinal quantitative lymphatic imaging. Generally, the generation of lymphatic vessels in tumor tissue is significantly correlated to lymph metastasis, while the potential remodeling and reimbursement routs of tumor-draining lymphatic vessels play a crucial role when peritumoral lymphatic drainage is impaired. Proulx et al. developed a range of bright NIR dye IRDye coupled with PEG (from 10 to 40 kDa) to observe the contraction of lymphatic vessels and distant lymphatic metastasis of tumor in real time. Compared with previous lymphangiography studies, these tracers dynamically described the functional status of collecting lymphatic vessels and draining LNs in vivo. (Fig. 8a–c). In order to investigate the relationship between the extent of anterior lymph node metastasis and lymphatic drainage, B16-F10-luc2 cell line and 4T1 cells were used to establish orthotopic tumor models. The increased lymphatic flow in tumor tissue and metastasis in popliteal lymph nodes were observed. In addition, the dysfunction of lymphatic flow caused by the growing metastatic tumor in LN changed the original pathways of lymphatic drainage and led to metastasize distantly (Fig. 8d) [124]. This concept may have major implications for the mapping and resection of peritumoral lymph nodes in clinical radical surgical treatment [127–129].

**Fig. 9:**
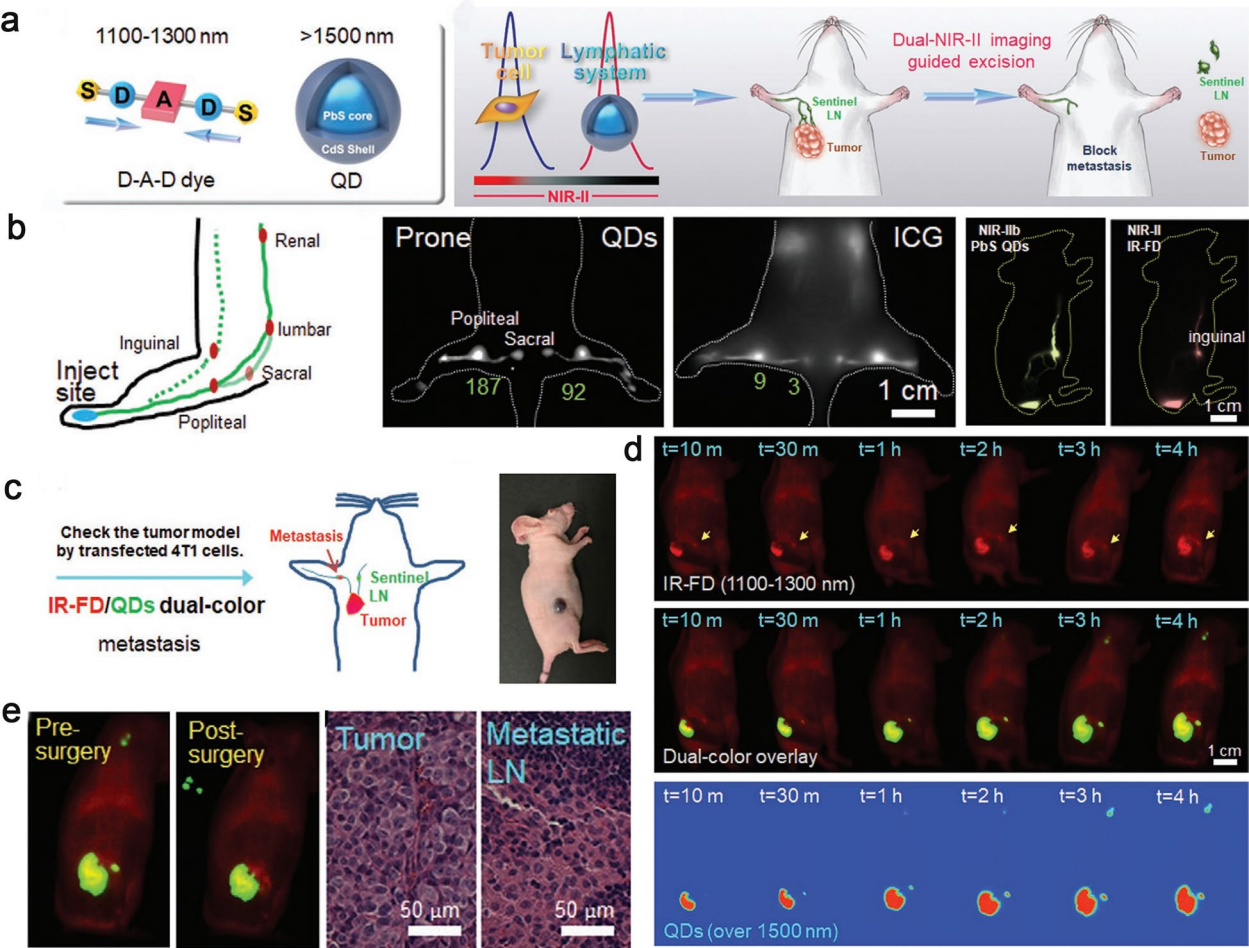
**a** General structure of dual NIR-II probes and NIR-II imaging-guided sentinel lymph nodes resection. **b** Schematic illustration of dual NIR II fluorescent dye injection and lymphatic drainage. **c** The illustration of dual-color imaging-guided surgery. **d** The different channels images displayed signal accumulation of IR-FD and QDs after administration of subcutaneous injection in tumor bearing mouse. **e** Dual NIR-II imaging guided pre- and post-surgery of sentinel LN excision. Reproduced with permission from [134]. Copyright 2020, Wiley–VCH Verlag GmbH & Co. KGaA, Weinheim

Compared with the conventional imaging at NIR-I (700–1000 nm), NIR-II imaging (1000–1700 nm) has become a promising non-invasive visualization method of deep lymphoid system with reduced tissue scattering and lower background [130–133]. Recent studies have shown that ICG also has the NIR-II imaging capability because of its extended long emission tail at NIR-II region [134, 135]. Chen et al*.* innovatively applied two regions of NIR-II (NIR-IIa and NIR-IIb) imaging modes to assist tumor resection and SLN biopsy. They reported a dual NIR-II fluorescence nanoplatforms containing a bright primary tumor-seeking D-A-D dye (IR-FD) with NIR-IIa (1100–1300 nm) imaging and a ultrabright PbS/CdS core–shell QDs with dense polymer coating for imaging tumor-metastasis SLNs at the NIR-IIb (> 1500 nm). T cell specific ligands were further attached to the surface of QDs to increase their retention capacity in SLNs, and PEG was linked to the D-A-D dye to increase the size, preventing self-aggregation and improving the water solubility. More importantly, this optimized D-A-D system dye exhibited extremely high quantum yield (up to 6.0%) and performed excellent imaging capability in vivo (Fig. 9) [134].

**Fig. 10:**
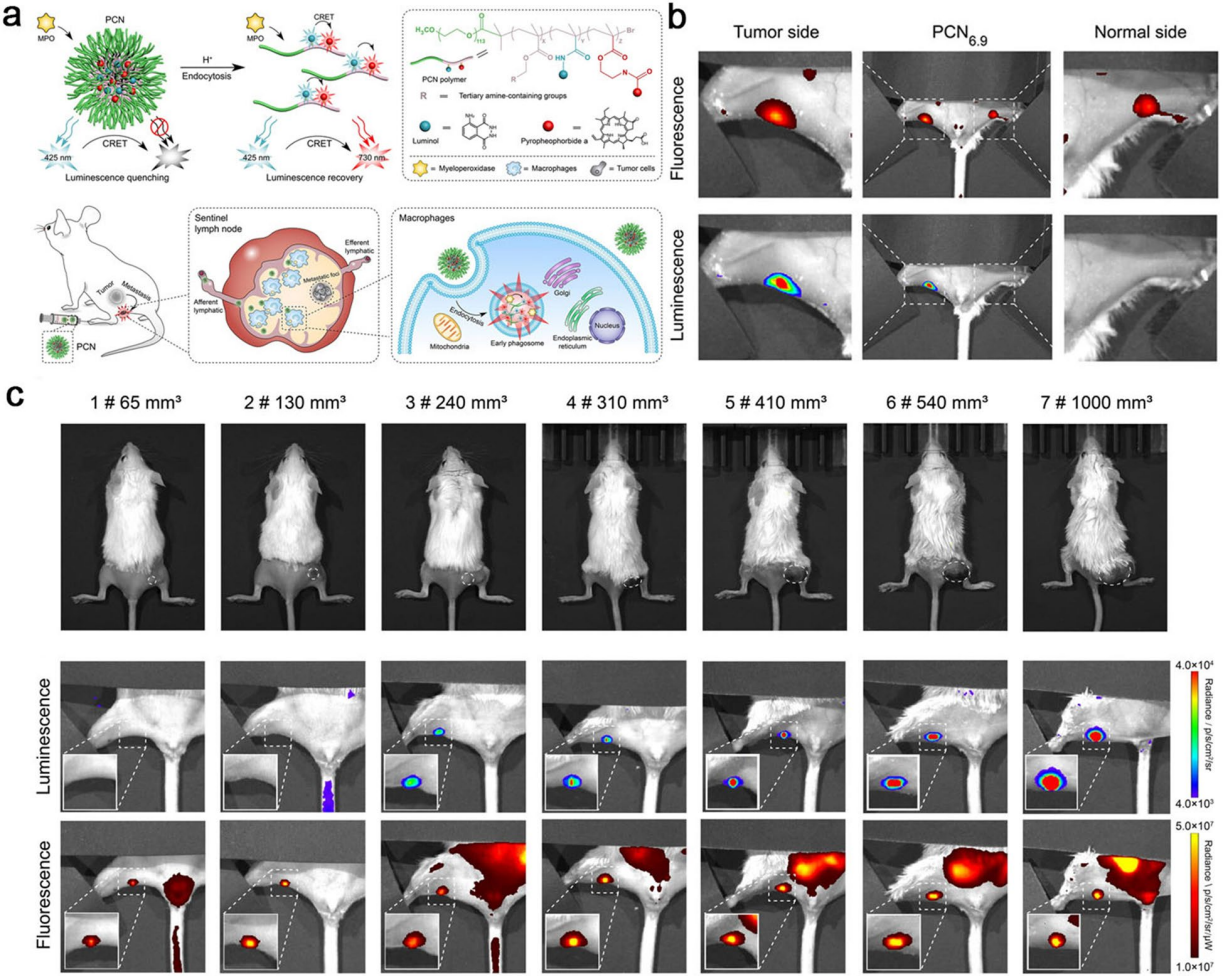
**a** The schematic of pH-amplified self-illuminating PCN for detecting sentinel lymph nodes with tumor metastasis in vivo*.* **b** The images of fluorescence imaging (up) and luminescence imaging (down) in vivo at tumor and normal sides after subcutaneously injected with PCN_6.9_. **c** Representative images of PCN identify metastasis lymph nodes through luminescence and fluorescence in the process of tumor growth. Top: appearance images of different stages of 4T1-GFP subcutaneous tumors. Middle and bottom: Luminescence and fluorescence images of the metastasis lymph nodes in vivo corresponding to the top. Reproduced with permission from [136]. Copyright 2021, Wiley–VCH Verlag GmbH & Co. KGaA, Weinheim

Matrix metalloproteinase (MMP) secretion is highly associated with tumor invasion and metastasis. Therefore, sensitive and accurate monitoring the changes of MMP secretion can indicate small metastatic tumors in SLNs and distant tissues. Liu et al*.* developed a cell membrane-anchored ratiometric upconversion nanomaterial, UCNPs-Cy3/Pep-QSY7/Ab, to visualise the MMP secretion in situ, and further applied in imaging metastatic SLNs. Cy3 and MMP2 substrate peptide labelled by QSY7 quencher (Pep-QSY7) were decorated on the surface of UCNPs. The UCNPs emission at 540 nm was quenched by QSY7 due to Cy3-mediated luminescence resonance energy transfer (LRET) process. Epidermal growth factor antibody (Anti-EGFR) was conjugated to UCNPs via a PEG44 linker for specific anchoring on the membrane of tumor cells. When MMP2 was secreted by tumor cells, Cy3 emission at 580 nm could be recovered upon MMP2-responsive Pep-QSY7 cleavage. So, the MMP2 secretion monitoring system anchored on the cell membrane achieved visualization of the MMP in metastatic SLNs in vivo [135]. Wang et al*.* developed a pH-responsive nanoformulation (PCN) by integrating chemiluminescence resonance energy transfer (CRET) and signal amplification strategy. When PCNs were drained into metastatic SLNs, they were phagocytosed by the tumor-associated macrophages and disintegrated in acidic phagosomes, then emit NIR fluorescence signal. Specifically, luminol and a NIR fluorescent probe pyropheophorbide a (PPa) were covalently conjugated to PCNs, luminol could precisely response to redundant myeloperoxidase (MPO) of macrophage in SLNs and emit bule luminescence, while PPa could reply the blue luminescence to emit NIR fluorescence through CRET. This strategy of integrating CRET and signal self-amplification could accurately identify the metastatic SLNs from benign ones and contribute to improve the efficacy and safety of surgery (Fig. 10) [136]. Additionally, cancer cells are known to produce an excess of free radicals, and the high levels of intrinsic ROS also render them more susceptible to extra oxide attack [137]. So, redox-sensitive nanomaterials based on the high concentrations of ROS in tumor cell are also a good option to accurate identification of the metastatic or inflammation SLNs. Song et al*.* synthesized several nanoplatforms for photoacoustic imaging, which showed promise in detecting SLNs precisely [140, 141]. As we have mentioned above, negatively charged nanomaterials as better fit to be transported into lymphatic vessels, while the positively charged nanoparticles are more efficacy endocytosed by cells. Stimuli-responsive charge-reversal nanoparticles (CRNPs) could sequence the two critical survival pathways together in lymphatic imaging. In generally, the charge-reversal nanomaterials are uncharged or negatively charged in a physiological setting and it allows the nanoparticles entry the lymph vessels rapidly. When the CRNPs are drained to the metastatic lymph nodes, they are endowed with positive charge in the metastatic tumor microenvironment and the enhanced endocytosis effect exhibits a durable imaging signal. However, it is a pity that the major research of this strategy is rarely focus on lymphatic imaging [142–145].

**Fig.11:**
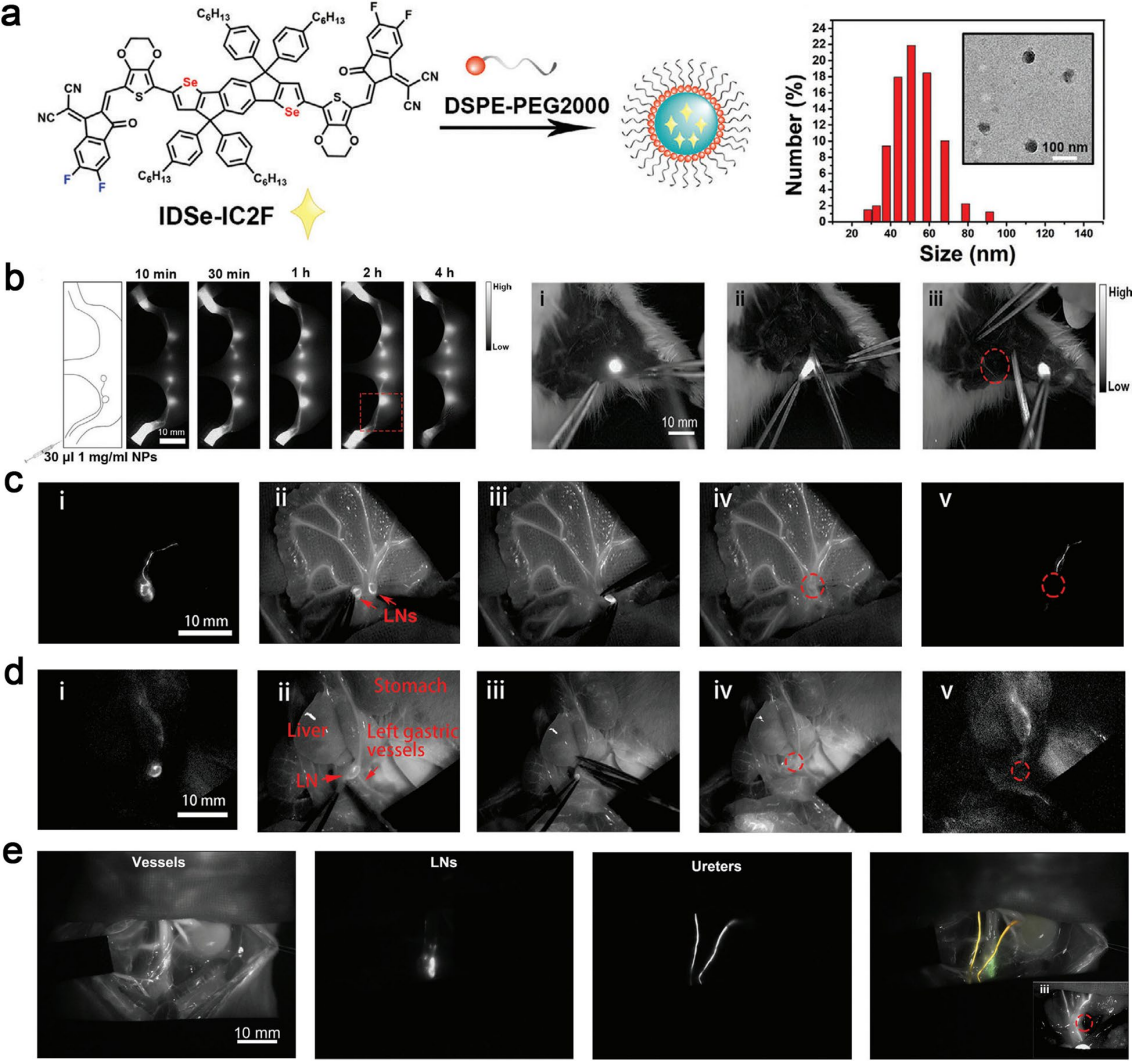
**a** The diagrammatic drawing of the fabrication process of IDSe-IC2F and the results of TEM and DLS. **b** NIR-II images of popliteal and sciatic LNs at different time points and NIR-II imaging-guided lymph node resection. **c** Precise positioning of mesenteric lymph nodes marked by IDSe-IC2F NPs and dual-channel fluorescence imaging guide lymphadenectomy on mesentery. **d** The location of gastric LNs labelled with IDSe-IC2F NPs and gastric vessels identified with TQ-BPN. i–iv: the process of resection of LNs closely to the left gastric artery. **e** Triple-channel NIR fluorescence images of paraaortic LNs (IDSe-IC2F), blood vessels (TQ-BPN NPs), and ureters (PEG-CSQDs) on rats with different excitation light sources (623 nm and 793 nm), the insert shows that triple-channel fluorescence imaging guide the lymphadenectomy beside the abdominal aorta. Reprint with the permission from [149]. Copyright 2021, Wiley–VCH Verlag GmbH & Co. KGaA, Weinheim

Different from traditional fluorescent probes suffering from the aggregation caused quenching (ACQ) phenomenon [144], a new type of fluorophore has been developed showing the opposite phenomenon, known as aggregation-induced emission (AIE) [147–150]. These luminescent groups become "brighter" in solid or aggregated state with higher quantum yields. Feng et al*.* successfully modified an extant NIR-I molecule whose emission extend to NIR-II (1200 nm) by converting heteroatomic sulfur to selenium in the receptor unit of D-A-D-type AIEgens. Nanoparticles named as L897 NPs encapsulating the NIR-II emissive AIEgens realized non-invasive lymphatic system imaging with high signal-to-background ratio. The fluorescent emission tail of this nanomaterial extended to 1200 nm and the quantum yield ascends to 5.8%. When L897 NPs were intradermally injected, the popliteal and sacral lymph nodes were quickly lit up and the lymphatic vessels between them were also clearly visualized [116]. Studies have shown that NIR-II fluorescence imaging was more suitable for multi-channel imaging because it has low background signal interference and less light scattering. Recently, a kind of excellent NIR-II fluorescence nanoparticles named as IDSe-IC2F was developed by Lin and co-workers, allowing imaging of lymphatic system with high spatiotemporal resolution. More interestingly, this novel complementary strategy that combined the multichannel fluorescence-assisted local lymph node cleaning strategy (cold) and NIR-II fluorescence-guided systemic photothermal ablation treatment (thermal) presented an effective therapeutic modality for cancer treatment (Fig. 11a, b). Nanoparticles with various fluorescence properties were used to describe lymph vessels and nods (IDSe-IC2F), blood vessels (TQ-BPN NPs), and ureters (PEG-CSQDs) at the same time, which contributed to reduce the incidence of side damage during lymph node dissection around the tumor and improved the safety of surgery (Fig. 11c–e). Furthermore, the photothermal effect of IDSe-IC2F was used to treat metastatic lymph nodes in dangerous areas. This "cold" and "hot" complementary strategy of multichannel NIR-II fluorescence imaging and imaging-guided photothermal therapy offered a feasible route for tumor precision theranostic [149].

### Photoacoustic imaging

Photoacoustic imaging (PAI) is a noninvasive and nonionizing imaging technology combining the advantages of high spatiotemporal resolution of optical imaging and excellent tissue penetration depth of ultrasound imaging [150]. Theoretically, it reduces the light scattering, breaks through the limit of optical imaging depth, thus achieving deeper tissue imaging up to several centimeters. This imaging modality holds unparalleled advantages in lymphatic system imaging in vivo [151]. The initial researches of lymphatic photoacoustic imaging mainly focus on SLNs imaging in animal models of breast cancer or melanoma [152]. Pan et al*.* designed a few gold nano-beacons with different sizes to noninvasively imaging SLN. The colloidal nano-platform with “soft” properties could be amenable to bio-elimination in the body and accumulate in lymph nodes [153]. As the existing technology evolving, further development of PAI gradually realized the functional detection of lymphatic vessels and lymph nodes. However, the gold element with high payload may lead to unsatisfactory lymphatic transport and uncomfortable intradermal injection. Recently, the naked carbon nanoparticles had been extracted from commercial honey for the first time, and a one-pot "green" technique was pursued to rapidly modify carbon nanoparticles with organic macromolecules (e.g., polysorbate, polyethylene glycol) under solvent free conditions. After subcutaneous injection, the photoacoustic imaging of the lymphatic system in the ROI could be portrayed quickly with a high resolution. In the wavelength range of 635–670 nm, the PA signal was nine times than that of blood, revealing the potential as an excellent candidate for PAI applications [154].

**Fig. 12:**
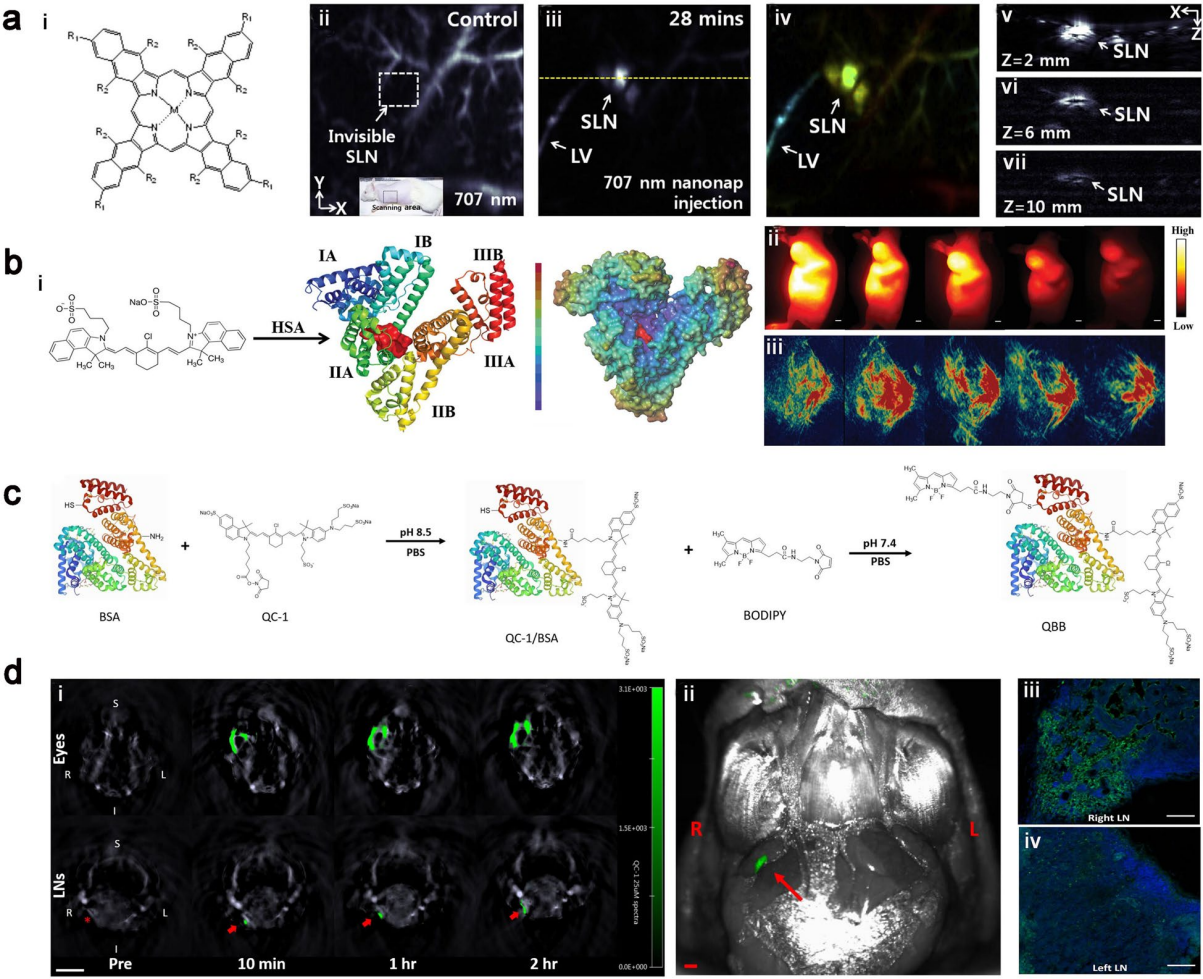
**a** i: Chemical structure of naphthalocyanines used in this research. ii–vii: the PA images of SLNs with injection of 707 nm nanonaps. Reproduced with permission [158]. Copyright 2015, Elsevier. **b** i: The simulation of the interaction of IR820 with HAS. ii, iii NIR imaging and PAI of tumors and biodistribution of IR820-BSA. Reprinted with permission from [160]. Copyright 2019, Wiley–VCH Verlag GmbH & Co. KGaA, Weinheim. **c** Synthesis of the hybrid QBB tracer. **d** i: The cross-sectional of PAI in the eyes (top row) and neck region (bottom row) after injection of QBB. ii: Fluorescent visualization of the neck after removal of skin and fat. Iii, iv: Confocal images indicate that QBB tracers lodged in the right neck LN (green signal). Reprinted with permission from [165]. Copyright 2021, Elsevier

Although nanostructures based on inorganic materials such as gold and carbon exhibit strong local surface plasmonic resonance, efficient targeting capabilities and excellent biocompatibility in preclinical animals [158–160]. The long-term safety remains the sticking point limiting their clinic trials. Kim et al*.* developed a new dual-color photoacoustic contrast formed from two biocompatible polyethylene blocks (refer to nanonaps) for lymphatic imaging in vivo. The nanonaps self-assembled from a couple of different near-infrared absorption naphthalocyanine dyes with the maximum absorbance at 707 nm and 860 nm. The absorbance of this nanoformulation in NIR region was nearly 100 times stronger than that of gold nanorods, an indicator that this nanomaterial was favorable for PAI. Apart from this, the lower toxicity, multi-color imaging capability and non-shifting spectral stability also allowed noninvasively lymphatic imaging. The bioconjugated nanonaps were successfully utilized for precise imaging of the lymphatic system and the individualized treatment of tumor (Fig. 12a) [158]. Albumin, a ubiquitous plasma protein in the human body, has been approved by FDA for several clinical applications, including Abraxane (albumin-bound paclitaxel) and albumin-bound docetaxel. Albumin containing hydrophobic domains could encapsulate NIR dyes through non-covalent interactions to improve the solubility, biocompatibility and utilization of theranostic cargoes [159]. The supramolecular bioconjugation of NIR dyes (e.g., ICG) with albumin is an efficacious platform for biomedical applications, such as fluorescence and photoacoustic imaging. Cheng et al*.* developed a supramolecular assembly of human serum albumin (HSA) complexed with a sulfonated organic dye IR820. Notably, the IR820-albumin complex could be easily synthesized using the FDA-approved ICG as a theranostic agent, showing excellent clinical translation potential (Fig. 12b) [160]. A 21-fold increase in fluorescence enhancement was realized through this strategy, which was suitable for NIR-II lymphatic imaging and PAI of SLN with a high resolution. The fluorescence of nanoformulations was contribute to validating the location of tracers in the process of PAI, assessing the intrinsic connection between nanoformulations and different organs [164, 165]. Compared with NIR fluorescent dyes, some non-fluorescent probes sometime exhibited relatively higher photostability and photoacoustic efficiency [166, 167]. It remains challenging to improve the photoacoustic sensitivity and resolution to fulfil the requires of lymphatic imaging. Yücel et al*.* described a hybrid photoacoustic tracer by conjugating albumin with a NIR non-fluorescent dye (QC-1) and a BODIPY-based fluorophore for photoacoustic lymphatic imaging. This novel hybrid nanoformulation QC-1/BSA/BODIPY (QBB) described lymphatic drainage from peripheral eyes to the neck with a minimum detectable concentration (2.5 μM) with high photostability. The imaging signal was quantitatively measured using PAI and the location of neck lymph node was visualized with fluorescence imaging after local injection of the prepared formulation QBB (Fig. 12c, d). This category of photoacoustic-fluorescent agents provided an intriguing method to enhance and refine existing clinical photoacoustic imaging techniques [165].

### Other imaging modalities

Contrast-enhanced ultrasound (CEUS) has been widely used in preclinical and clinical trials. It has been demonstrated that ultrasound microbubbles prefer to accumulate in sentinel lymph nodes, rather than the secondary or further lymph nodes, possibly due to the high affinity of coating materials to macrophages [166]. Numbers of submicron particles have been used for imaging of regional lymph nodes after subcutaneous injection [170–173]. It is generally believed that the optimal image size of the lymphatic system was found to be in the range of 5 to 100 nm. Interestingly, microbubbles with a diameter of several microns also seem to be able to pass through the peripheral lymphatic endothelium and colonize in the local lymph nodes to realize the identification of benign or malignant [169]. Despite their widespread applicability in tumor imaging and diagnosis, the low imaging resolution, slow subcutaneous migration rates, inaccessibility to deep retroperitoneum and other defects have limited their use in clinical lymphatic imaging.

**Fig. 13:**
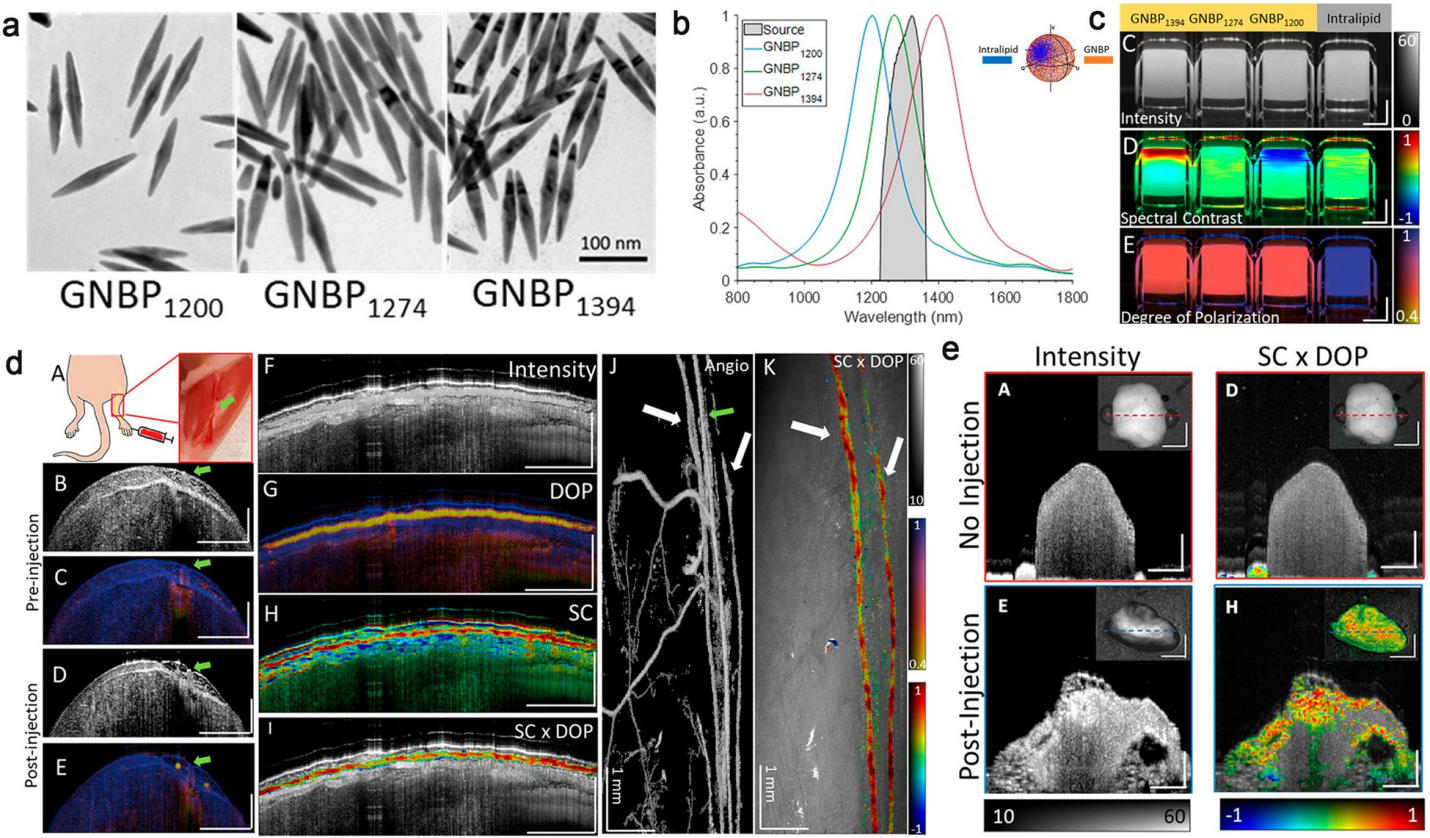
**a** TEM images of GNBPs. **b** Spectra of GNBPs. **c** Intensity, spectral contrast and degree of polarization images of GNBPs. **d** Schemic and representative images of GNBPs on the hind limb in mice. **e** Representative images of ex vivo popliteal lymph node before and after GNBP_1394_ injection. Reproduced from [172], Copyright 2021, American Chemical Society

Optical coherence tomography (OCT) is a label-free imaging technique in vivo which makes lymphatic vessels “visible” through the negative contrast of highly scattered surrounding tissues [171]. This technique allows rapid volume scanning, quantitative evaluation of the lymphatic function and three-dimensional imaging at the micron scale (5–20 μm). As a pioneer in complex dynamic imaging of living tissues, it has shown advantages in the visualization of lymphatic vessels in conjunctiva and skin. Regrettably, due to the little detectable change in the index of refraction in the NIR-II, the vast majority of small molecules and fluorescent probes lack the intrinsic OCT contrast. Development of novel imaging probes for OCT is favourable to visualize biomarkers concurrently with anatomical features at a depth of a few millimetres with a resolution of micrometer-scale. Furthermore, polarization-sensitive (PS)-OCT can provide additional information about tissue retardation and depolarization based on the polarization state of the detected light. Keahey et al*.* successfully achieved multiplexed mapping of lymph vessels and associated draining lymph nodes after subcutaneous injections of GNBPs by combing PS-OCT and spectral contrast (SC)-OCT measurements. This novel detection and multiplexing technique open a door to optical molecular imaging and enable to map lymphangiogenesis in tumors and metastatic SLNs (Fig. 13) [172].

### Multimodal imaging

**Fig. 14:**
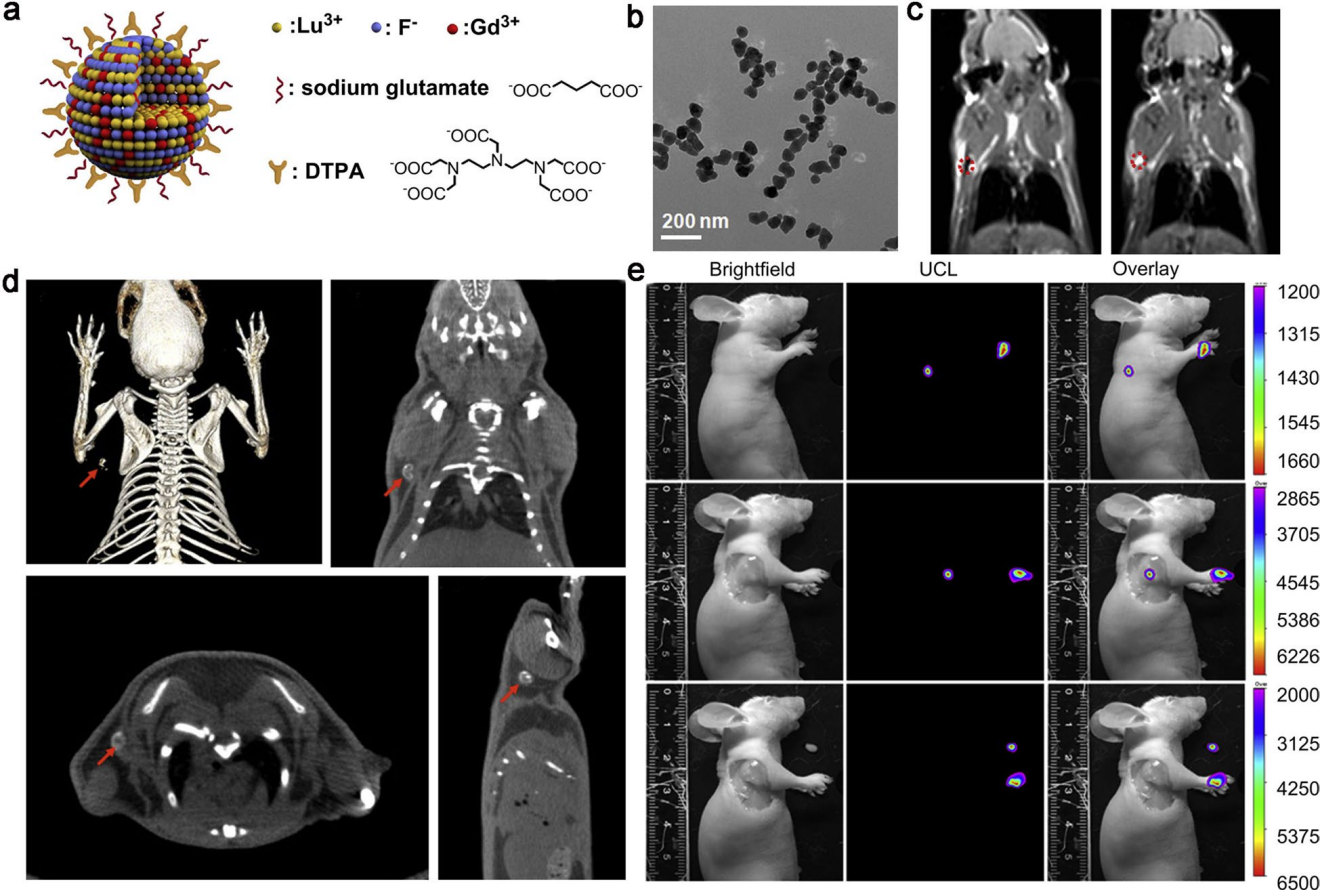
**a**, **b** Structure of the Lu-UCNPs and TEM image. **c** MRI images of lymph node at 0 and 30 min after intradermal injection of Lu-UCNPs. **d** Multimodal images (PET/CT/MRI) of lymph node after intradermal injection of Lu-UCNPs. **e** Lymph node images were detected in vivo, in situ and ex vivo after injection of Lu-UCNPs in nude mice. Reproduced with permission from [174]. Copyright 2012, Elsevier

In order to overcome the inherent defects of single imaging, the multimodal imaging emerged by integrating the merits of individual modalities. PET-CT is an effective way to diagnose lymphatic disorders, which combines the precise positioning of positron emission and high-resolution of tomography technology, and. With the maturation of multimodal imaging technologies, such as PET/CT, SPECT/CT, PET/MRI etc., various multifunctional imaging probes have been developed to realize synergistic lymphatic imaging. To compensate for the lack of imaging resolution in lymphoscintigraphy, Kobayashi et al*.* reported a multimodal and multicolor nanosized imaging agent, which coalesced ^111^In-labeled radionuclide and five-color optical dyes to visualize the local lymphatic drainage and detect SLNs. Radionuclides allowed absolute quantification and augmented penetration depth, while multicolor optical imaging described different drainage regions with superior spatiotemporal resolution [173]. Li et al*.* developed NaLuF_4_-based multifunctional upconversion nanoparticles doping with Gd^3+^, Yb^3+^, Er^3+^ and Tm^3+^. Lutetium (Lu) showed strong X-ray absorption for CT imaging, lanthanide performed excellent upconversion luminescence signal with unique properties, such as sharp emission lines, large stokes shifts, superior photostability, and non-blinking. Gd^3+^ provided outstanding T_1_-weighted MRI signal and relaxation. Studies had shown that the relaxivity parameter (r_1_) could reach as low as 2.273 s^−1^ mM^−1^, which was half of commercial T_1_-enhanced MRI agents while the signals acquired was higher than most of the reported Gd-doped nanoparticles (Fig. 14) [174].

In 2001, PET/CT was officially introduced into clinic use for the first time. Subsequently, commercial PET/MR was successfully installed and put into use in 2010. The multimodal imaging technology with PET as the fundamental pillar has advanced by giant leaps in the past 10 years. Meanwhile, the development of new multimodal probes based on PET possesses outstanding benefits, greatly promoting scientists to develop novel contrast agents. The most representative example is the Cornell dots (C dots), inorganic nanoprobe integrating PET and fluorescence dual-mode imaging modalities [175]. Bradbury et al. conducted the first human imaging trial using C Dots as multimodal imaging contrast. Combining the pivotal advantages of PET and NIR optical imaging, these C-dots were successfully applied in imaging of human melanoma, which brought the dawn to multimodal imaging of clinical lymphatic system [176].

**Fig. 15:**
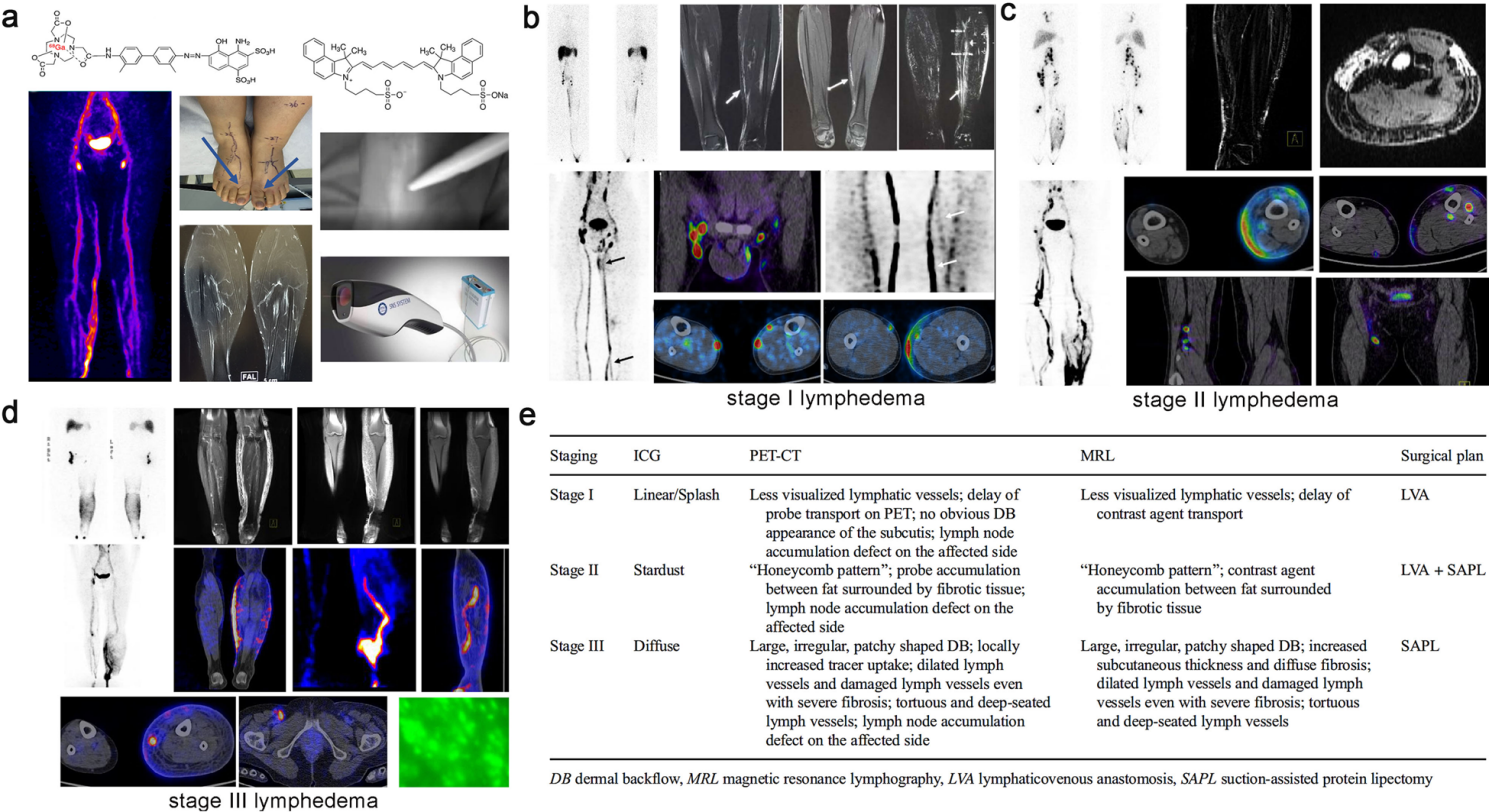
**a** The process of ^68^Ga-NEB combined with ICG for PET/MR/ICG lymphangiography. **b**–**d** Lymphatic imaging of both lower limbs in a patient with stage I, II, III lymphedema. Multimodal imaging details (PET, MRI and FI) could provide more evidence to judge the severity of lymphedema disease. **e** Multimodal imaging findings and the formulations of surgical plan based on imaging data in patients with lymphedema. Reproduced with permission from [177]. Copyright 2017, Springer Berlin Heidelberg

With the popularity of microsurgical techniques in the treatment of limbs lymphedema, the “road map” of lymphatic system is becoming increasingly important in preoperative clinical staging and intraoperative navigation. In order to better promote the basic research to clinic, Chen et al. used ^68^ Ga-NEB to evaluate the feasibility of combining PET/MRL with FL (ICG) in rapidly visualizing lymphatic system and preoperative staging in lymphedema. The results proved that ^68^ Ga-NEB could provide detailed three-dimensional structures of central and peripheral lymphatic system with higher temporal resolution. Moreover, the picture of impaired lymphatic vessels was clearly revealed by combining PET/MRL and fluorescence lymphangiography. These multimodal images had important implications for evaluating lymphedema severity and staging damaged lymph vessels, thus contributing to make an individualized treatment plan (Fig. 15) [177].

**Fig. 16:**
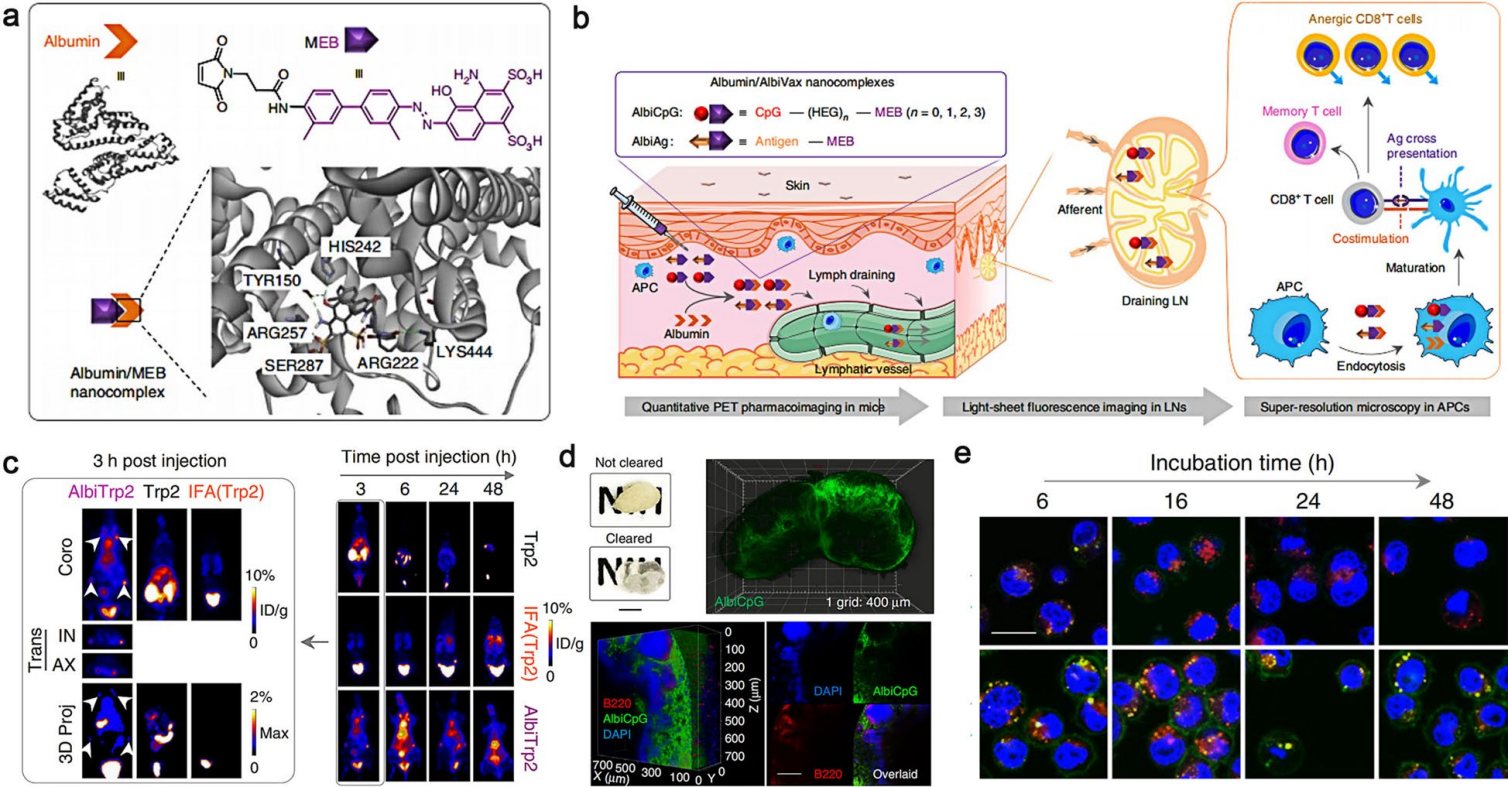
**a** Schematic structure of albumin/MEB nanoformulations. **b** The illustration of multimodal imaging strategies including whole-body imaging, organ/tissue imaging, and cellular imaging. **c** Representative PET images of AlbiVax in vivo. **d** Light sheet fluorescence microscopy images depict 3D intranodal distribution of AlbiVax. **e** Representative confocal microscopy images of AlbiVax in APCs. Reprinted from [178]

The lymphatic vessels provide channels for lymphocytes, antigen presenting cells (APCs) and antigens traffic from tissues to draining lymph nodes, where the lymphocytes execute the function of immune monitoring. From this perspective, more functional information about the lymphatic system will be found by using multimodal imaging strategies to track immune cells in the lymph at different levels. Chen et al*.* reported a promising clinical translation albumin/AlbiVax nanoformulations via conjugating molecular vaccines with EB into albuminbinding vaccines (AlbiVax), which can self-assemble in vivo with desirable biological functions (Fig. 16). There was almost 100-fold more efficient co-delivery of CpG and agents to lymph nodes than benchmark incomplete Freund’s adjuvant (IFA) through the quantitative evaluation of PET pharmacoimaging, light-sheet fluorescence and super-resolution microscopies. This multimodal imaging strategy at different levels including whole-body, intravital organ and cell provided the potential of exquisitely quantitative the lymphatic immune function [178].

## Summaries and prospects

In this review, a series of fantastic research about the design strategy of lymphatic nanoformulations and lymphatic imaging modalities have been summarized. The individual modalities based on various creative nanomaterials discussed above had express high-quality information of lymphatic drainage. As an ideal lymphatic contrast agent, the following characteristics should be taken into consideration:$= 1 \backslash *\mathrm{ roman }$ (i) the optimal scale (5–100 nm); (ii) a suitable modification based on different imaging mechanisms and purposes including size, surface charge, target sites etc.; (iii) long-term biosafety concern about the excessive use of organic solvents and the immune response of nanomaterials retained in vivo; (iv) increased utilisation of biological components such as haemoglobin and albumin. These strategies of “hitchhiking” and “self-assembly in vivo” have significant potentials in incorporation into novel biomaterials.

To better access the function of lymphatic, it is imperative to integrate multiple advanced lymphatic imaging modalities. Multimodal imaging technology with PET as the fundamental pillar has been widely developed in clinical practice in addition to lymphatic imaging, which may be related to lacking of fewer available clinical lymphatic contrast agents and the specific anatomy of peripheral lymphatic vessels. Represented by near-infrared fluorescence and photoacoustic imaging have brought light to the mentioned predicament above. It is envisioned that PET-CT (MRI)/optical multimodal imaging is promising become the best combination in clinical diagnosis and treatment in the future.

There are a lot of important factors need to be considered in precise imaging of LNs including the locations of LNs (superficial or peritoneal), the characteristics of LNs (immunity or tumor metastatic), the injection practise of contrast agents (intravenous or subcutaneous injection) and so on. In order to specifically deliver imaging agents to targeting LNs, a major consideration emphasizes the difference of individual LNs microenvironment. In particularly, there are several options available for targeting the corresponding LNs such as the specific antigenic peptides presented on the surface of target cells, the lower pH or higher level of ROS/GSH in tumor/inflammation extracellular microenvironment, the aggregation of tumor-associated immune cells, excessive cytokine or enzyme release associated with tumor/inflammation etc. These factors described above can be used as stimuli during the preparation of nanoformulations for imaging LNs precisely.

Surgery remains the mainstay of therapy for multiple primary diseases. Clinicians in the worldwide are now faced with the dilemma: how to avoid the iatrogenic injury and meanwhile make sure radical treatment? Imaging-guided accurate intraoperation navigation provides a feasible solution to address the problem. Given that the lymphatic system is characterized by colorless transparency and minor diameter, the lymphatic imaging with high spatiotemporal resolution could present accurate information of lymph nodes and lymphatic vessels in tumor radical resection and lymphatico-venous anastomosis, maximally shortening the operation time, improving the process efficiency and avoiding iatrogenic injury. Certainly, synthesizing nanomaterials with excellent properties and choosing a suitable multimodal imaging method are the first and key step to achieve these goals. Over the past decades, the research on lymphatic system is far less advanced than other systems owing to lacking of powerful means to visualize it comprehensively. As the interesting of lymphatic imaging continues evolve, it is imperative to look for innovative nanomaterials to overcome the obstacle by taking full advantage of individual element with distinct characteristics and eventually achieve clinical translation. We anticipate that an overall impressive description of the central-peripheral-superficial lymphatic system can be depicted at one time in the not-too-distant future and the lymphatic system is bound to return to the spotlight.

## Data Availability

Not applicable.

## Abbreviations

LECs: Lymphatic endothelial cells
LSG: Lymphoscintigraphy
SPECT: Single photon emission computed tomography
FI: Fluorescence imaging
PET: Positron emission tomography
MRI: Magnetic resonance imaging
MRL: Magnetic resonance lymphography
PAI: Photoacoustic imaging
SPIO: Superparamagnetic iron oxide
SLN: Sentinel Lymph Node
ICG: Indocyanine green
ECM: Extracellular matrix
LYVE-1: Lymphatic Vessel Endothelial Receptor-1
^18^F-FDG: ^18^F-Fludeoxyglucose
EB: Evans Blue
CLI: Cerenkov luminescence imaging
PAMAM: Polyamidoamine
CND: Carboxylated nanodiamond
SSFP: Steady-state free precession
NIR-II: Near-infrared II
QDs: Quantum dots
D-A-D: Donor–acceptor–donor
ACQ: Aggregation caused quenching
AIE: Aggregation-induced luminescence
ROI: Region of interest
HAS: Human serum albumin
CEUS: Contrast-enhanced ultrasound
OCT: Optical coherence tomography
C dots: Cornell dots
DB: Dermal backflow
LVA: Lymphaticovenous anastomosis
SAPL: Suction-assisted protein vessels
APCs: Antigen presenting cells
